# Supplementary material for: The UFM1 E3 ligase recognizes and releases 60S ribosomes from ER translocons
Source: Nature. 2024 Feb 21;627(8003):437–44. doi: 10.1038/s41586-024-07093-w (PMC10937380; doi:10.1038/s41586-024-07093-w)
Supplement: Supplementary file 1 — Supplementary Fig. 1 (summary of XL-MS data) and Supplementary Table 1. [file 41586_2024_7093_MOESM1_ESM.pdf]

nature portfolio

https://doi.org/10.1038/s41586-024-07093-w

Supplementary information

The UFM1 E3 ligase recognizes and releases 60S ribosomes from ER translocons

In the format provided by the authors and unedited

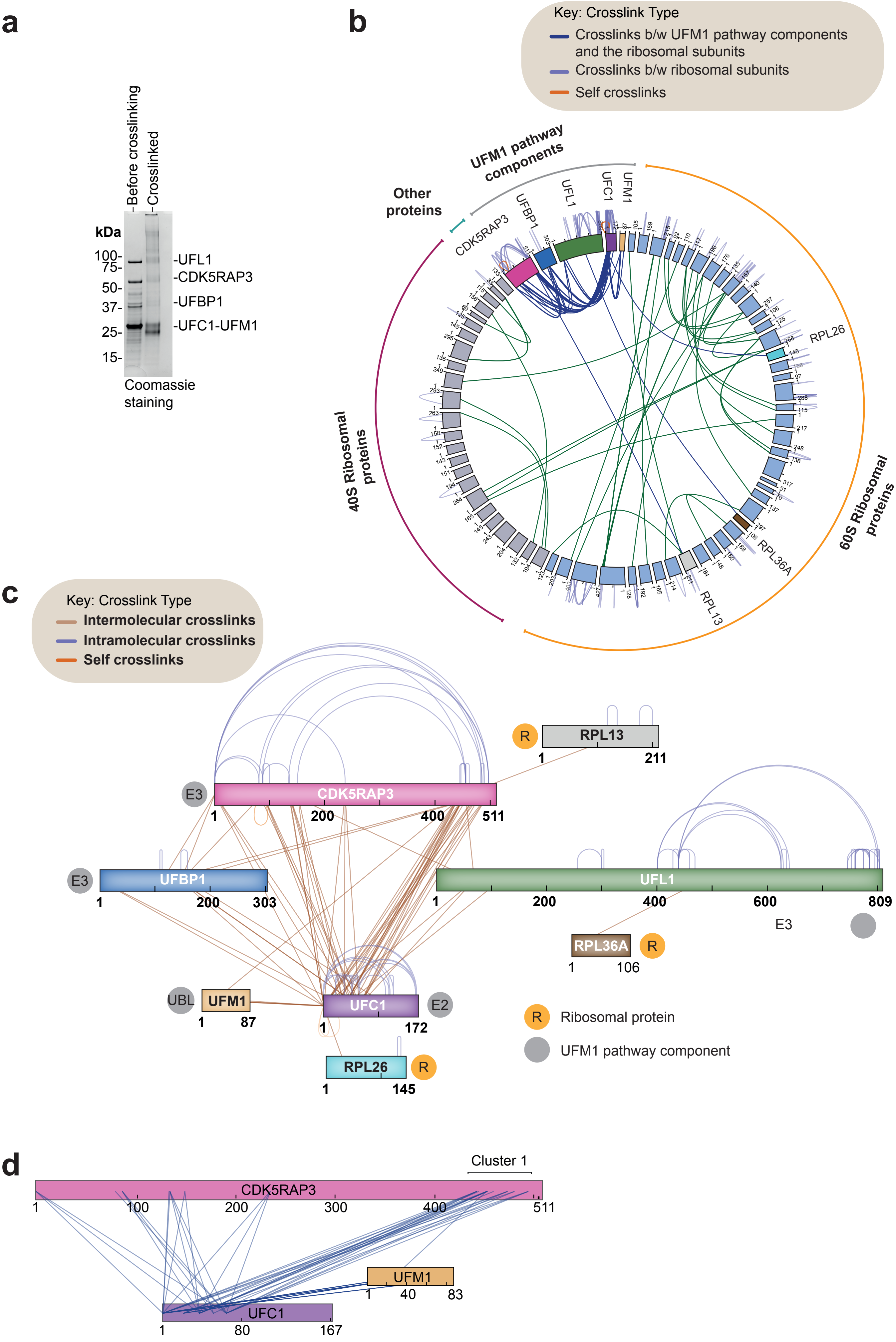

a
Before crosslinking
Crosslinked
kDa
100-
75-
50-
37-
25-
15-
-UFL1
-CDK5RAP3
-UFBP1
-UFC1-UFM1
Coomassie staining
b
Key: Crosslink Type
Crosslinks b/w UFM1 pathway components and the ribosomal subunits
Crosslinks b/w ribosomal subunits
Self crosslinks
UFM1 pathway components
Other proteins
CDK5RAP3
UFBP1
UFL1
UFC1
UFM1
RPL26
RPL36A
RPL13
40S Ribosomal proteins
60S Ribosomal proteins
c
Key: Crosslink Type
Intermolecular crosslinks
Intramolecular crosslinks
Self crosslinks
RPL13
1
211
E3
CDK5RAP3
1
200
400
511
E3
UFBP1
1
200
303
UFL1
1
200
400
600
809
E3
RPL36A
1
106
UBL
UFM1
1
87
UFC1
E2
1
172
RPL26
1
145
R
Ribosomal protein
UFM1 pathway component
d
Cluster 1
CDK5RAP3
1
100
200
300
400
511
UFM1
1
40
83
UFC1
1
80
167

Supplementary Figure 1 Summary of crosslinking-MS data

a, Coomassie-stained SDS-PAGE gel analysing formation of UREL-ribosome complexes in the presence and absence of the crosslinker disuccinimidyl dibutyric urea (DSBU). The protein complexes were incubated with DSBU for 30 min at 23 °C. The crosslinking reaction was quenched by adding excess Tris (pH 8) and run on a 4-12% SDS-PAGE gel followed by Coomassie staining. **b**, Circular plot showing crosslinks formed between UREL, UFC1-UFM1 and ribosomal subunits. The ligase components specifically interact with the 60S ribosomal subunits. **c**, Crosslinks between UREL, UFC1-UFM1 and the ribosomal subunits derived from above are shown to highlight the interaction network. **d,** Schematic showing intermolecular crosslinks between UFC1, UFM1 and CDK5RAP3 identified by crosslinking mass spectrometry (XL-MS).

Supplementary Table 1. Details of antibodies and recombinant proteins

Antibodies and dilution used		
Anti-UFM1 (1:1000)	Abcam	ab109305
Anti-UBA5 (1:1000)	Universal Biologicals	A304-115A-T
Anti-UFC1 (1:1000)	Abcam	ab189252
Anti-UFL1 (1:1000)	Abcam	ab227506
Anti-CDK5RAP3 (1:1000)	Bethyl Laboratories	A300-870A
Anti-rabbit IgG, HRP-linked Antibody (1:10,000)	CST	70745
IRDye 800CW anti-Rabbit (1:10,000)	Li-COR	926-32211
IRDye 680CW anti-Rabbit (1:10,000)	Li-COR	926-68071
Anti-UFBP1 (DDRGK1) (1:1000)	Proteintech	21445-1-AP
Anti-UFSP2 (1:1000)	Abcam	ab192597
Anti-RPL26 (1:1000)	Abcam	ab59567
Anti-ERp72 (1:1500)	CST	5033T
Anti-GAPDH (1:5000)	Abcam	ab8245
Anti-RPS10 (1:1000)	Abcam	ab151550
Anti-RPL10A (1:5000)	Abcam	ab174318
Anti-ODR4 (1:1000)	Abcam	Ab121495
**Recombinant proteins**		
6xHis-TEV-UFL1/StrepII-UFBP1 29-C K193A	This Study	DU76181
6xHis-TEV-UFL1 /StrepII-UFBP1 29-C F196A	This Study	DU76182
6xHis-TEV-UFL1 /StrepII-UFBP1 29-C K193D	This Study	DU76187
6xHis-TEV-UFL1 StrepII-UFBP1 29-C V198R	This Study	DU76188
6xHis-TEV-UFL1 /StrepII-UFBP1 29-C F196R	This Study	DU76190
6xHis-TEV-UFL1 /StrepII-UFBP1 29-C	Peter et al[3]	DU63479
6xHis-3C-UFM1 1-83	Peter et al.[3]	DU59441
6xHis-3C-UFM1 1-83 K69R	Peter et al.[3]	DU59446
6xHis-3C-UFM1 1-83 K0	Peter et al.[3]	DU59472
GST-3C-UFBP1 178-204	This Study	DU75665
GST-3C-UFC1	This Study	DU73281
GST-3C-UFC1 L32R	This Study	DU75492
GST-3C-UFC1 I40R	This Study	DU75493
GST-3C-UFC1 D50A	This Study	DU75504

GST-3C-UFC1 C116K	This Study	DU73986
6xHis-TEV-UFM1 1-83	This Study	DU73256
6xHis-UFL1 1-179/UFBP1 207-C	This Study	DU72757
6xHis-UFL1 1-179 I8R/UFBP1 207-C	This Study	DU75507
6xHis-UFL1 1-179 L11R/UFBP1 207-C	This Study	DU75508
6xHis-UFL1 1-179 F15R/UFBP1 207-C	This Study	DU75509
6xHis-UFL1 1-179 Q19R/UFBP1 207-C	This Study	DU75505
6xHis-UFL1 1-179/UFBP1 207-C R265A	This Study	DU75506
6xHis-UFL1 22-179/Strep-UFBP1 207-C	This Study	DU75115
6xHis-TEV-UFL1 1-179/UFBP1 174-C	This Study	DU75543
6xHis-TEV-UFL1 1-179 del21-24/UFBP1 174-C	This Study	DU75672
6xHis-TEV-UFL1 1-179 del25-28/UFBP1 174-C	This Study	DU75673
6xHis-TEV-UFL1 1-179/UFC1 C116K-UFBP1 204-C	This Study	DU75485
6xHis-3C-UBA5	Peter et. al.[3]	DU32106
8xHis-MBP-3C-CDK5RAP3	Peter et. al.[3]	DU59674
