# Supplementary material for: The UFM1 E3 ligase recognizes and releases 60S ribosomes from ER translocons
Source: Nature. 2024 Feb 21;627(8003):437–44. doi: 10.1038/s41586-024-07093-w (PMC10937380; doi:10.1038/s41586-024-07093-w)
Supplement: Supplementary file 2 — Reporting Summary [file 41586_2024_7093_MOESM2_ESM.pdf]

nature portfolio

Corresponding author(s): Elton Zeqiraj, Yogesh Kulathu

Last updated by author(s): Dec 15, 2023

Reporting Summary

Nature Portfolio wishes to improve the reproducibility of the work that we publish. This form provides structure for consistency and transparency in reporting. For further information on Nature Portfolio policies, see our Editorial Policies and the Editorial Policy Checklist.

Statistics

For all statistical analyses, confirm that the following items are present in the figure legend, table legend, main text, or Methods section.

n/a	Confirmed	
☐	☒	The exact sample size (*n*) for each experimental group/condition, given as a discrete number and unit of measurement
☐	☒	A statement on whether measurements were taken from distinct samples or whether the same sample was measured repeatedly
☐	☒	The statistical test(s) used AND whether they are one- or two-sided<br>*Only common tests should be described solely by name; describe more complex techniques in the Methods section.*
☒	☐	A description of all covariates tested
☒	☐	A description of any assumptions or corrections, such as tests of normality and adjustment for multiple comparisons
☐	☒	A full description of the statistical parameters including central tendency (e.g. means) or other basic estimates (e.g. regression coefficient) AND variation (e.g. standard deviation) or associated estimates of uncertainty (e.g. confidence intervals)
☐	☒	For null hypothesis testing, the test statistic (e.g. *F*, *t*, *r*) with confidence intervals, effect sizes, degrees of freedom and *P* value noted<br>*Give P values as exact values whenever suitable.*
☒	☐	For Bayesian analysis, information on the choice of priors and Markov chain Monte Carlo settings
☒	☐	For hierarchical and complex designs, identification of the appropriate level for tests and full reporting of outcomes
☒	☐	Estimates of effect sizes (e.g. Cohen's *d*, Pearson's *r*), indicating how they were calculated

Our web collection on statistics for biologists contains articles on many of the points above.

Software and code

Policy information about availability of computer code

Data collection	Yes described and referenced in Methods section.
Data analysis	Yes, described and referenced in Methods section. Crystallography data was processed with the autoPROC suite (including XDS62, Pointless, Aimless, CCP4 and STARANISO or Xia2 and DIALS. Molecular replacement - PHASER. Refinement and model building- REFMAC and Coot (CCP4i2 suite). Cryo-EM data processing - MOTIONCOR2 and CTFFIND4.1 (RELION-3.1), crYOLO-1.6.1, cryoSPARC-3.2, cryoSPARC-4.2.1, cryoDRGN-3.2.0, cryoSPARC-4.2.1 3DFlex. Model building, map sharpening and refinement - Phenix-1.2.1, DeepEMhancer, Coot-0.9.8.1. Visualization - UCSF ChimeraX-1.2.5 software

For manuscripts utilizing custom algorithms or software that are central to the research but not yet described in published literature, software must be made available to editors and reviewers. We strongly encourage code deposition in a community repository (e.g. GitHub). See the Nature Portfolio guidelines for submitting code & software for further information.

Data

Policy information about availability of data

All manuscripts must include a data availability statement. This statement should provide the following information, where applicable:

- Accession codes, unique identifiers, or web links for publicly available datasets
- A description of any restrictions on data availability
- For clinical datasets or third party data, please ensure that the statement adheres to our policy

The cryo-EM coordinates have been deposited to the Protein Data Bank under accession codes 8QFD (ligase bound 60S) and 8QFC (ligase only). Cryo-EM maps have been deposited to the Electron Microscopy Data Bank under accession codes EMD-18382 (ligase bound 60S) and EMD-18381 (ligase only).
X-ray structure factors and associated models have been deposited to the Protein Data Bank under accession codes 8COD and 8BZR. The Raw DSBU XL-MS data and the LC-MS/MS analysis of ribosome UFMylation have been deposited to the ProteomeXchange Consortium via the PRIDE partner repository with the dataset identifiers PXD046990 and PXD046991, respectively.

Research involving human participants, their data, or biological material

Policy information about studies with human participants or human data. See also policy information about sex, gender (identity/presentation), and sexual orientation and race, ethnicity and racism.

Reporting on sex and gender	N/A
Reporting on race, ethnicity, or other socially relevant groupings	N/A
Population characteristics	N/A
Recruitment	N/A
Ethics oversight	N/A

Note that full information on the approval of the study protocol must also be provided in the manuscript.

Field-specific reporting

Please select the one below that is the best fit for your research. If you are not sure, read the appropriate sections before making your selection.

☒ Life sciences ☐ Behavioural & social sciences ☐ Ecological, evolutionary & environmental sciences

For a reference copy of the document with all sections, see nature.com/documents/nr-reporting-summary-flat.pdf

Life sciences study design

All studies must disclose on these points even when the disclosure is negative.

Sample size	Where necessary, we have repeated experiments and the number of biological and technical replicates are indicated in the figure legends. In our experience for cell-based assays in cell lines (n>3) and in vitro assays using purified proteins (n>2), reproducibility with these numbers are sufficient.
Data exclusions	No data was excluded
Replication	In general, we aimed for sufficient technical and biological replicates as indicated in each figure legend to ensure robust reproducibility of observations
Randomization	No treatments were performed on different biological samples where human bias could be a factor, randomization was not applicable.
Blinding	No treatments were performed on different biological samples where human bias could be a factor, blinding was not applicable

Reporting for specific materials, systems and methods

We require information from authors about some types of materials, experimental systems and methods used in many studies. Here, indicate whether each material, system or method listed is relevant to your study. If you are not sure if a list item applies to your research, read the appropriate section before selecting a response.

Materials & experimental systems

n/a	Involved in the study
☐	☒ Antibodies
☐	☒ Eukaryotic cell lines
☒	☐ Palaeontology and archaeology
☒	☐ Animals and other organisms
☒	☐ Clinical data
☒	☐ Dual use research of concern
☒	☐ Plants

Methods

n/a	Involved in the study
☒	☐ ChIP-seq
☒	☐ Flow cytometry
☒	☐ MRI-based neuroimaging

Antibodies

Antibodies used

Anti-UFM1 Abcam ab109305
Anti-UBA5 Universal Biologicals A304-115A-T
Anti-UFC1 Abcam ab189252
Anti-UFL1 Abcam ab227506
Anti-UFBP1 Abcam ab99121
Anti-CDK5RAP3 Bethyl Laboratories A300-870A
Anti-RPL26 Bethyl laboratories A300-686A-M
Anti-rabbit IgG, HRP-linked Antibody CST 70745
IRDye 800CW anti-Rabbit Li-COR 926-32211
IRDye 680CW anti-Rabbit Li-COR 926-68071
Anti-UFBP1 (DDRGK1) Proteintech 21445-1-AP
Anti-CDK5RAP3 Proteintech 11007-1-AP
Anti-UFSP2 Abcam ab192597
Anti-RPL26 Abcam ab59567
Anti-ERp72 CST 5033T
Anti-GAPDH Abcam ab8245
Anti-RPS10 Abcam ab151550
Anti-RPL10A Abcam ab174318
Anti-SEC61-b (gift from R.Hegde, MRC LMB Cambridge)

Validation

Since most of the antibodies listed here were used in biochemical assays, the specificity of the antibody was established by having conditions where the protein was not present and a signal was not detected at the corresponding molecular weight by immunoblotting. Specificity statements are also available on the manufacturer's website:
Anti-UFM1 – See specificity statement from the manufacturer's website - https://www.abcam.com/products/primary-antibodies/ufm1-antibody-epr42642-ab109305.html

Anti-UBA5 - See specificity statement from the manufacturer's website - https://www.thermofisher.com/antibody/product/UBA5-Antibody-Polyclonal/A304-115A-T

Anti-UFC1 - See specificity statement from manufacturer's website. - https://www.abcam.com/products/primary-antibodies/ufc1-antibody-epr15014-102-ab189252.html

Anti-UFL1 - See specificity statement from manufacturer's website – https://www.abcam.com/products/primary-antibodies/ufl1-antibody-n-terminal-ab227506.html

Anti-CDK5RAP3 – See specificity statement from manufacturer's website – https://www.thermofisher.com/antibody/product/CDK5RAP3-Antibody-Polyclonal/A300-870A

Anti-DDRGK1 - See specificity statement from manufacturer's website - https://www.ptglab.com/products/DDRGK1-Antibody-21445-1-AP.htm

Anti-UFSP2 - See specificity statement from manufacturer's website - https://www.abcam.com/products/primary-antibodies/ufsp2-antibody-ep13424-49-ab192597.html

Anti-RPL26 - See specificity statement from manufacturer's website - https://www.abcam.com/products/primary-antibodies/rpl26-antibody-ab59567.html
For the anti-Sec61b antibody, it was validated in the publication from the Hegde lab. for example - doi:10.1083/jcb.200210095

Eukaryotic cell lines

Policy information about cell lines and Sex and Gender in Research

Cell line source(s)	Flp-In T-REx HEK293 cells (Invitrogen; R78007)
Authentication	None

Mycoplasma contamination	Cell lines were routinely tested for mycoplasma and were negative for mycoplasma contamination
Commonly misidentified lines (See ICLAC register)	Not used
