# Supplementary material for: The UFM1 E3 ligase recognizes and releases 60S ribosomes from ER translocons
Source: Nature. 2024 Feb 21;627(8003):437–44. doi: 10.1038/s41586-024-07093-w (PMC10937380; doi:10.1038/s41586-024-07093-w)
Supplement: Supplementary file 6 — Source Data Figs. 1 and 4. [file 41586_2024_7093_MOESM6_ESM.pdf]

Source Data Figure 1: Uncropped gel scans from Main Figures

a, Immunoblots (from Figure 1a) analysing UFMylation of 60S and 80S ribosomes obtained from sucrose density gradient of membrane fractions from WT Flp-In 293 T-REx cells. Three different gels were run and respective membranes were probed for UFM1, RPL26 and RPS6, respectively.

b, Immunoblots (from Figure 4c) showing *in vitro* UFMylation of 60S ribosomes in the presence of indicated UFC1 mutants that are defective in E3 binding. Samples were run on two separate SDS-PAGE gels and transferred onto two different membranes. Membrane 1 was probed for RPL26 and membrane 2 was probed for UFC1, respectively.

C, Coomassie-stained SDS-PAGE gel under non-reducing conditions (from Figure 4d) showing lysine discharge assays in the presence of $E3_{mUU}$ bearing truncations (UFL1∆21-24 and UFL1∆25-28) in the hinge region.

d, Coomassie-stained SDS-PAGE gels (from Figure 4e) showing SEC fractions of UFL1-Δα1/UFBP1/UFC1-UFM1, UFL1-Δα1/UFBP1/CDK5RAP3 and UFL1-Δα1/UFBP1/CDK5RAP3/UFC1-UFM1.

e, Immunoblots from Figure 5b analysing 60S ribosomal fractions from sucrose density gradient fractionation of parental WT and CDK5RAP3 KO cells. Samples were run on three different SDS-PAGE gels. Membrane 1 was probed for SEC61β and membrane 2 was probed for RPL26. Membrane 3 was first probed for RPL10a and then re-probed for UFM1.

Figure 1a

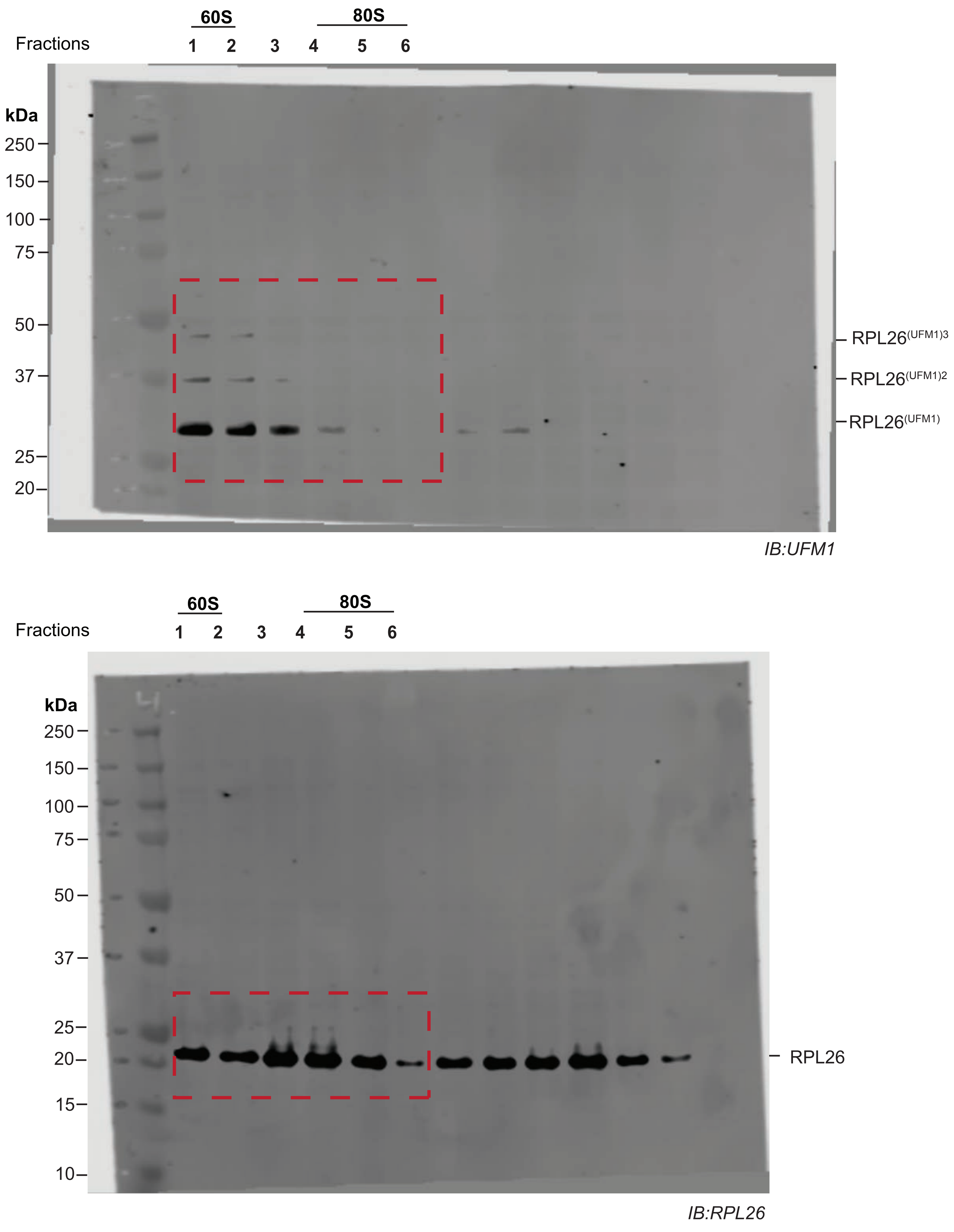
60S
80S
Fractions
1
2
3
4
5
6
kDa
250
150
100
75
50
37
25
20
RPL26(UFM1)3
RPL26(UFM1)2
RPL26(UFM1)
IB:UFM1
60S
80S
Fractions
1
2
3
4
5
6
kDa
250
150
100
75
50
37
25
20
15
10
RPL26
IB:RPL26

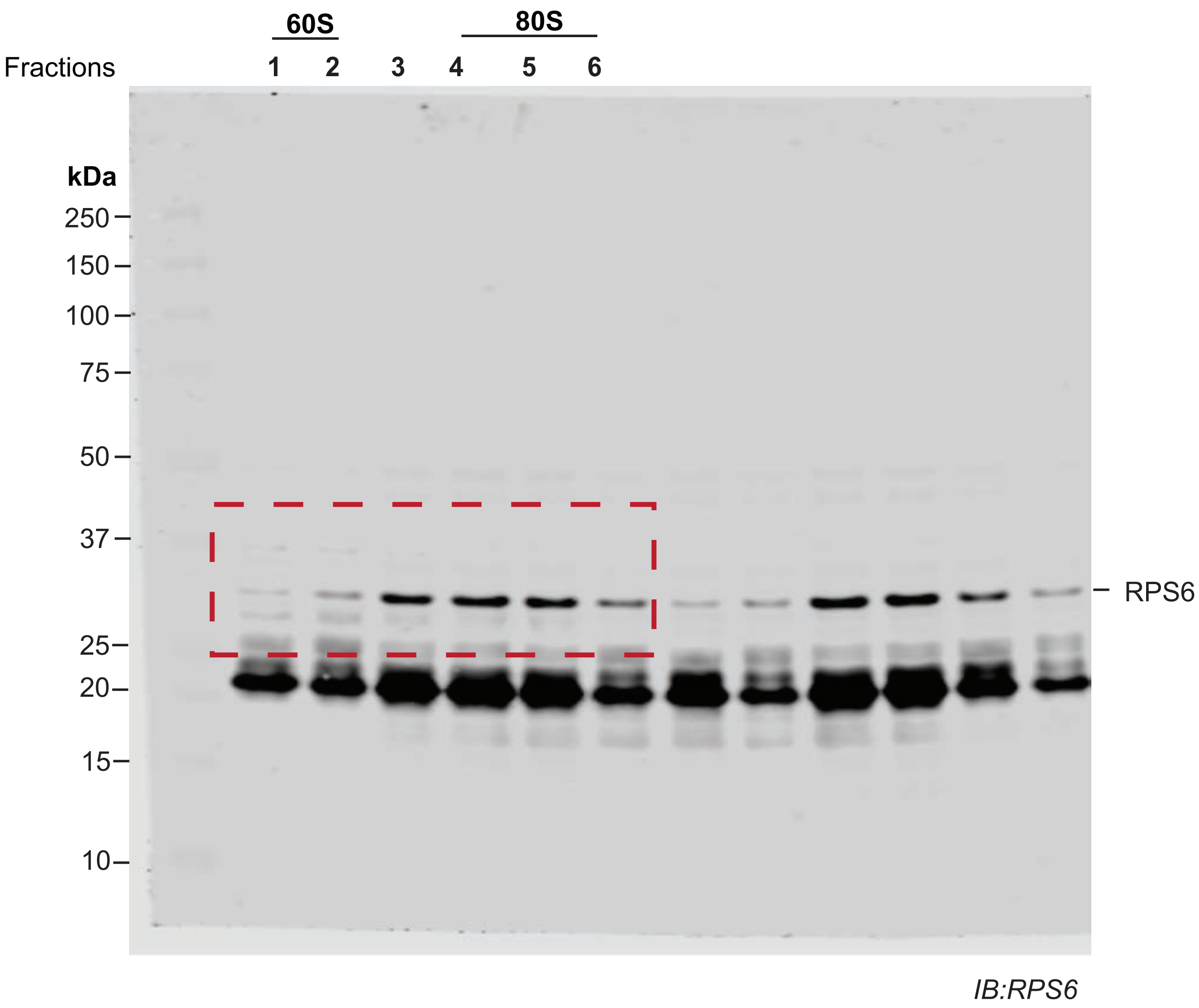
60S
80S
Fractions
1
2
3
4
5
6
kDa
250
150
100
75
50
37
25
20
15
10
RPS6
IB:RPS6

b

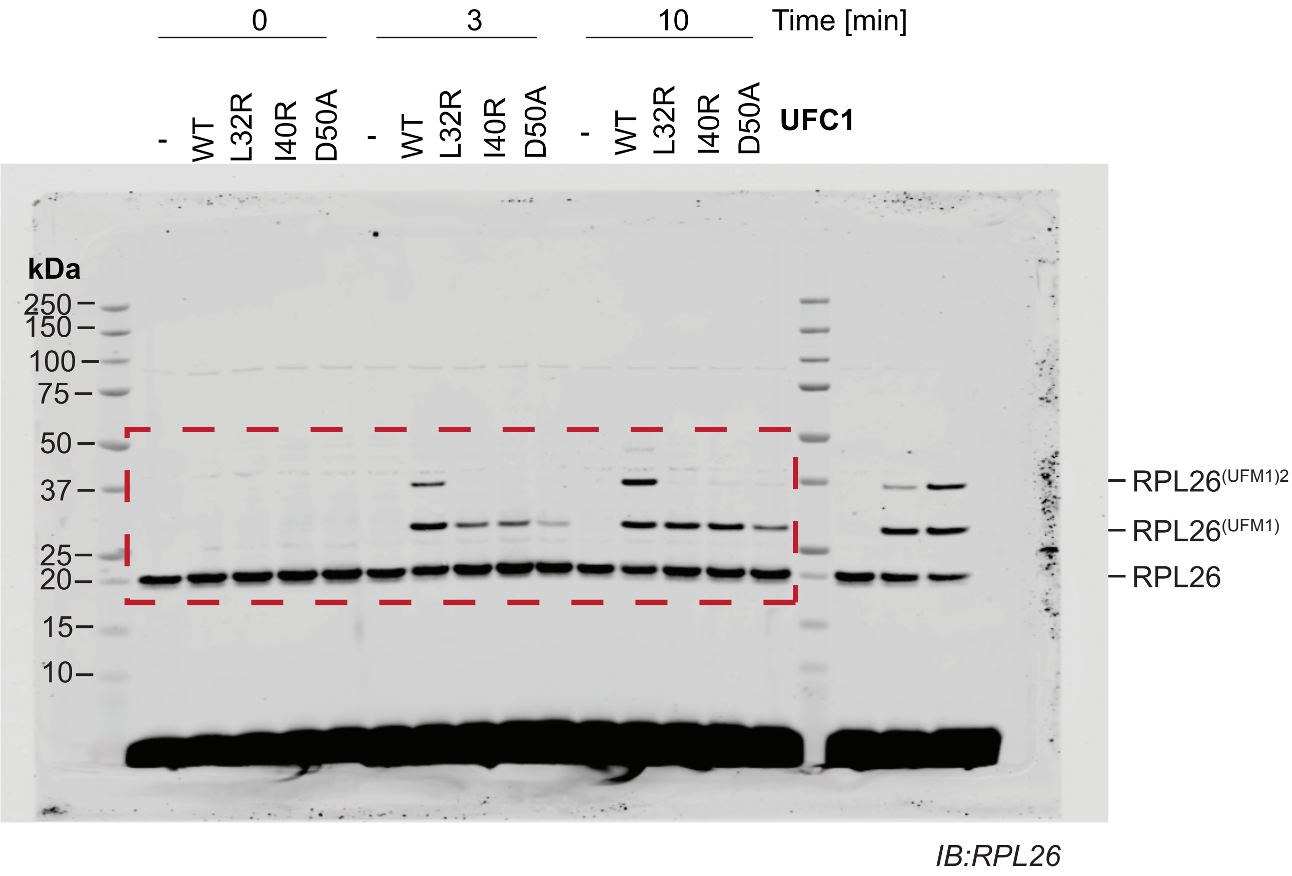
0
3
10
Time [min]
-
WT
L32R
I40R
D50A
-
WT
L32R
I40R
D50A
-
WT
L32R
I40R
D50A
UFC1
kDa
250
150
100
75
50
37
25
20
15
10
RPL26(UFM1)2
RPL26(UFM1)
RPL26
IB:RPL26

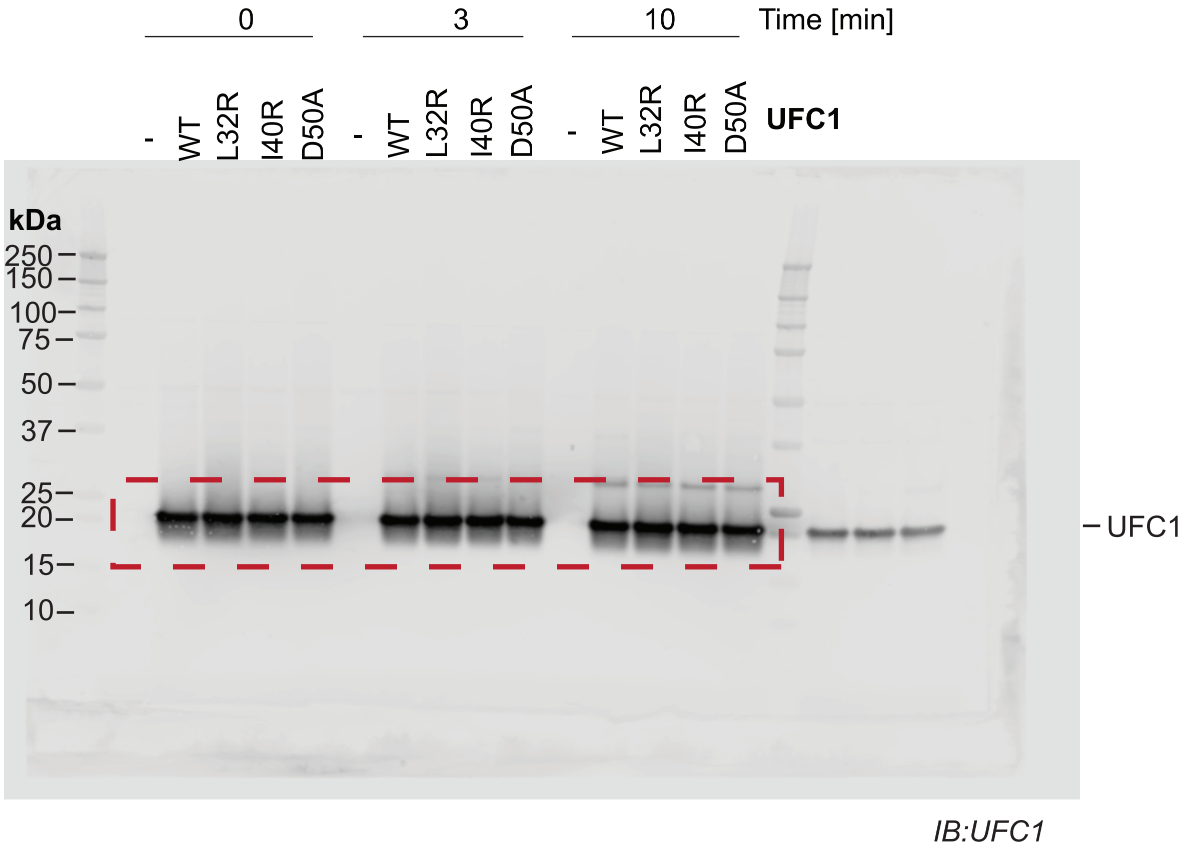
0
3
10
Time [min]
-
WT
L32R
I40R
D50A
UFC1
kDa
250
150
100
75
50
37
25
20
15
10
UFC1
IB:UFC1

c

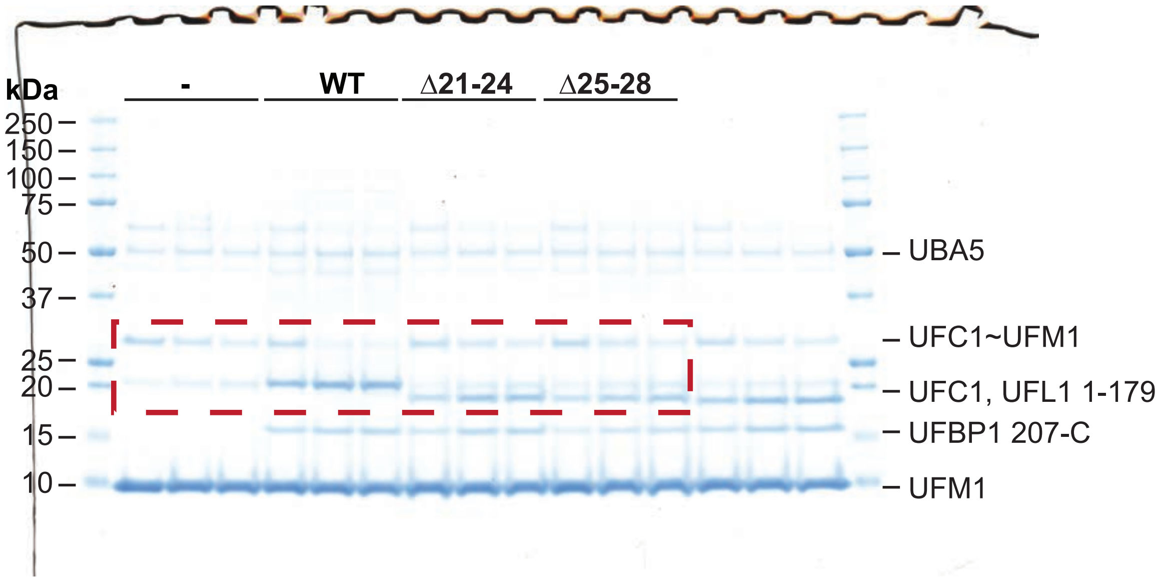
kDa
-
WT
Δ21-24
Δ25-28
250
150
100
75
50
37
25
20
15
10
UBA5
UFC1~UFM1
UFC1, UFL1 1-179
UFBP1 207-C
UFM1

d

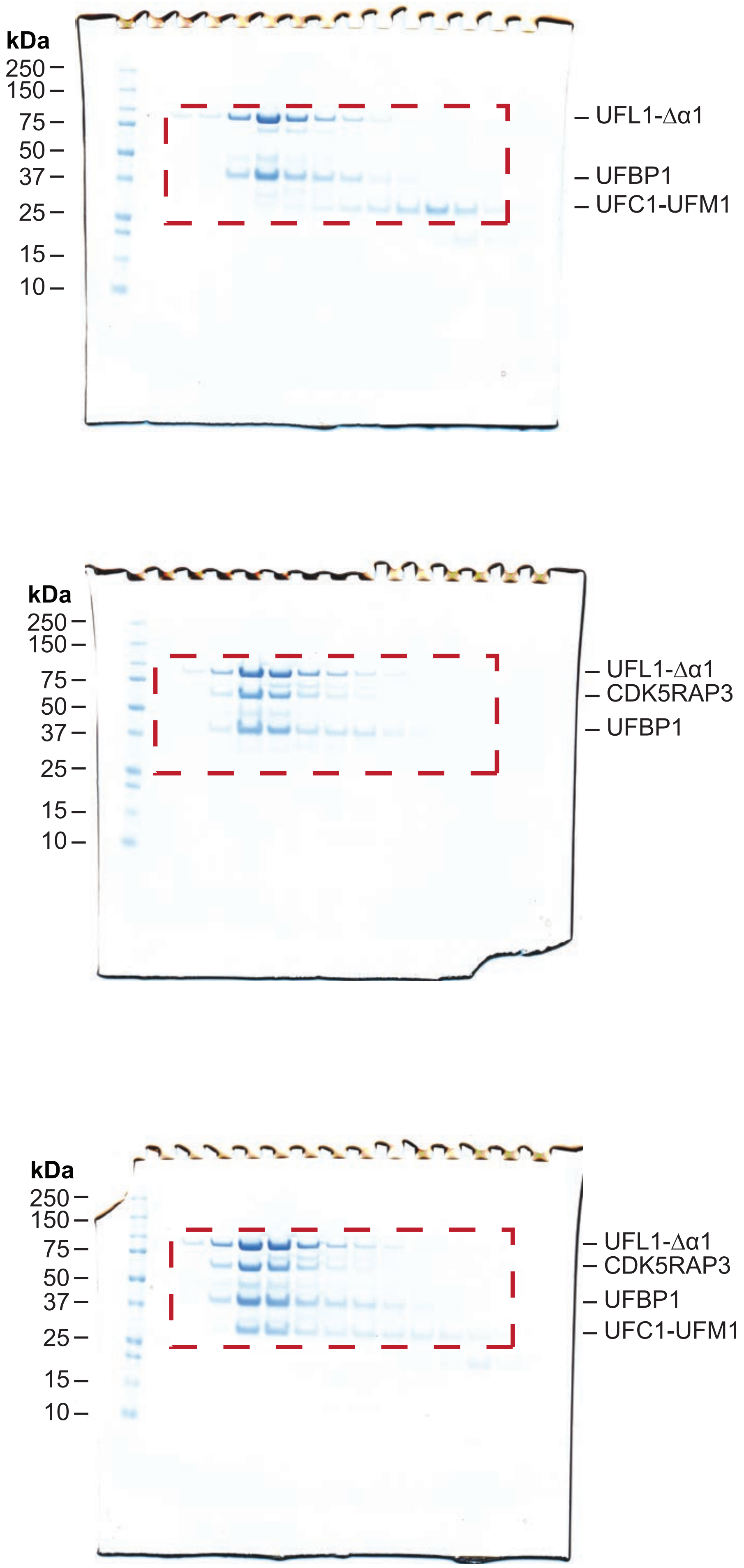
kDa
250
150
75
50
37
25
15
10
UFL1-Δα1
UFBP1
UFC1-UFM1
kDa
250
150
75
50
37
25
15
10
UFL1-Δα1
CDK5RAP3
UFBP1
kDa
250
150
75
50
37
25
15
10
UFL1-Δα1
CDK5RAP3
UFBP1
UFC1-UFM1

e

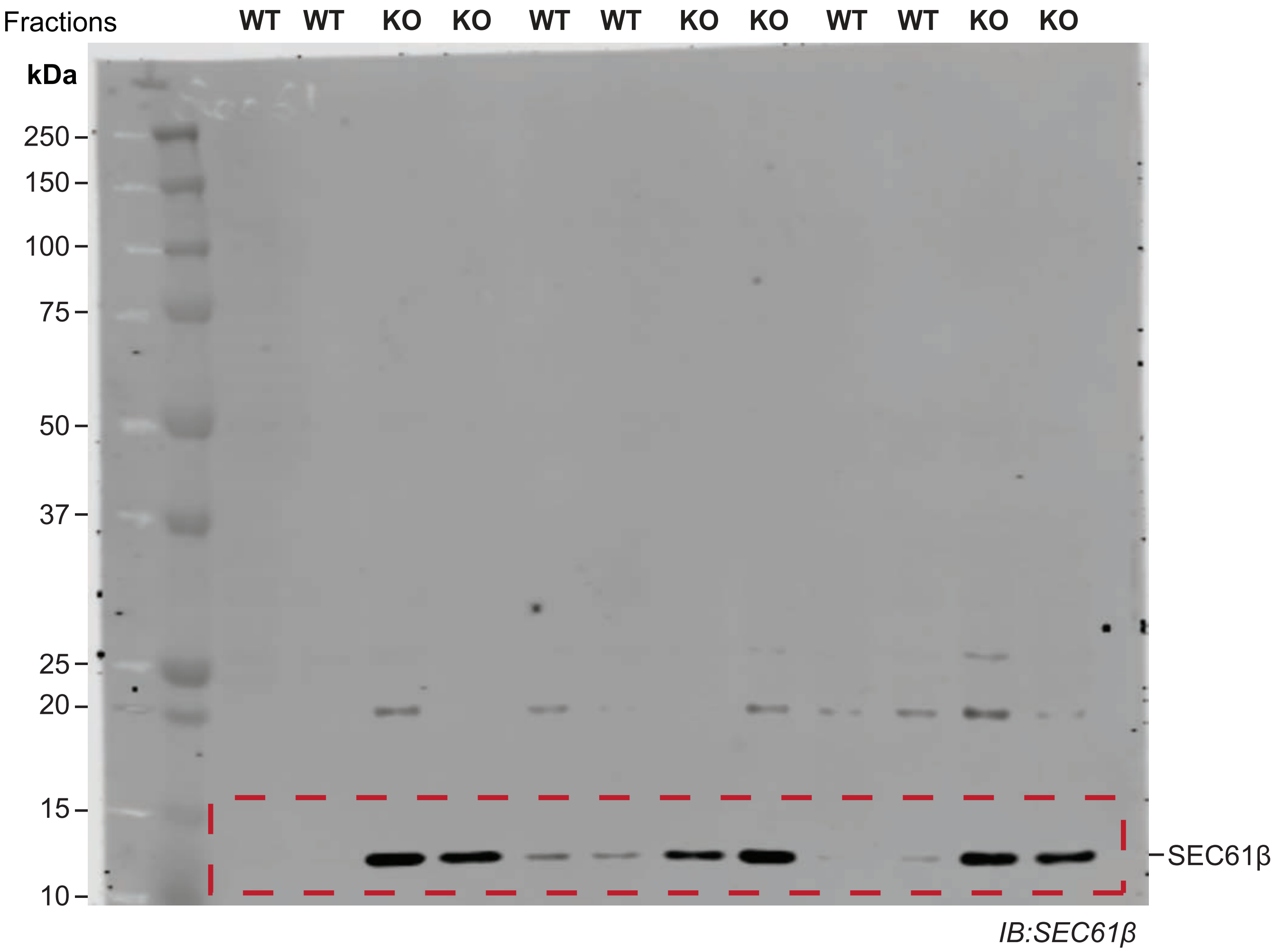
Fractions
WT WT KO KO WT WT KO KO WT WT KO KO
kDa
250
150
100
75
50
37
25
20
15
10
SEC61β
IB:SEC61β

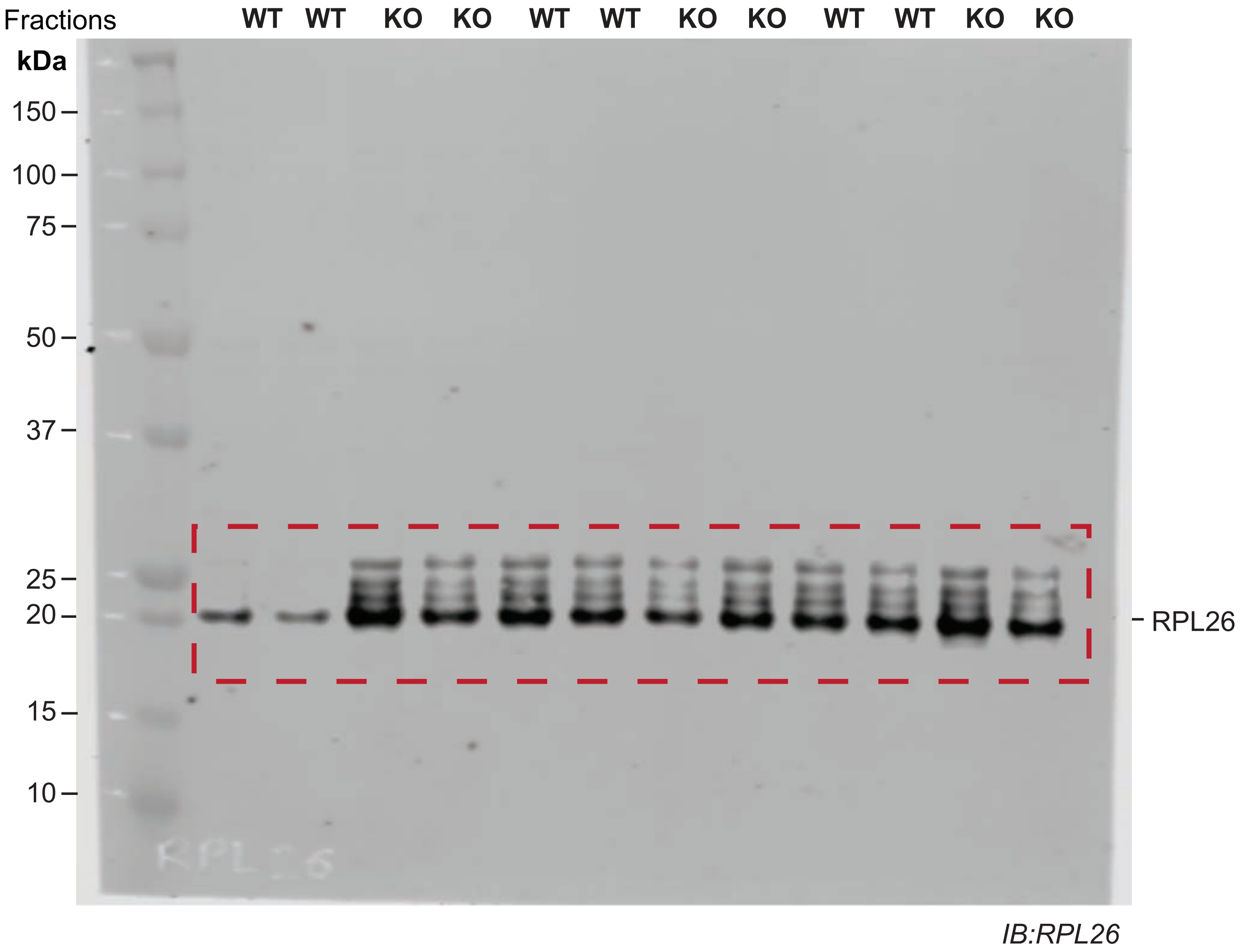
Fractions
WT WT KO KO WT WT KO KO WT WT KO KO
kDa
150
100
75
50
37
25
20
15
10
RPL26
IB:RPL26

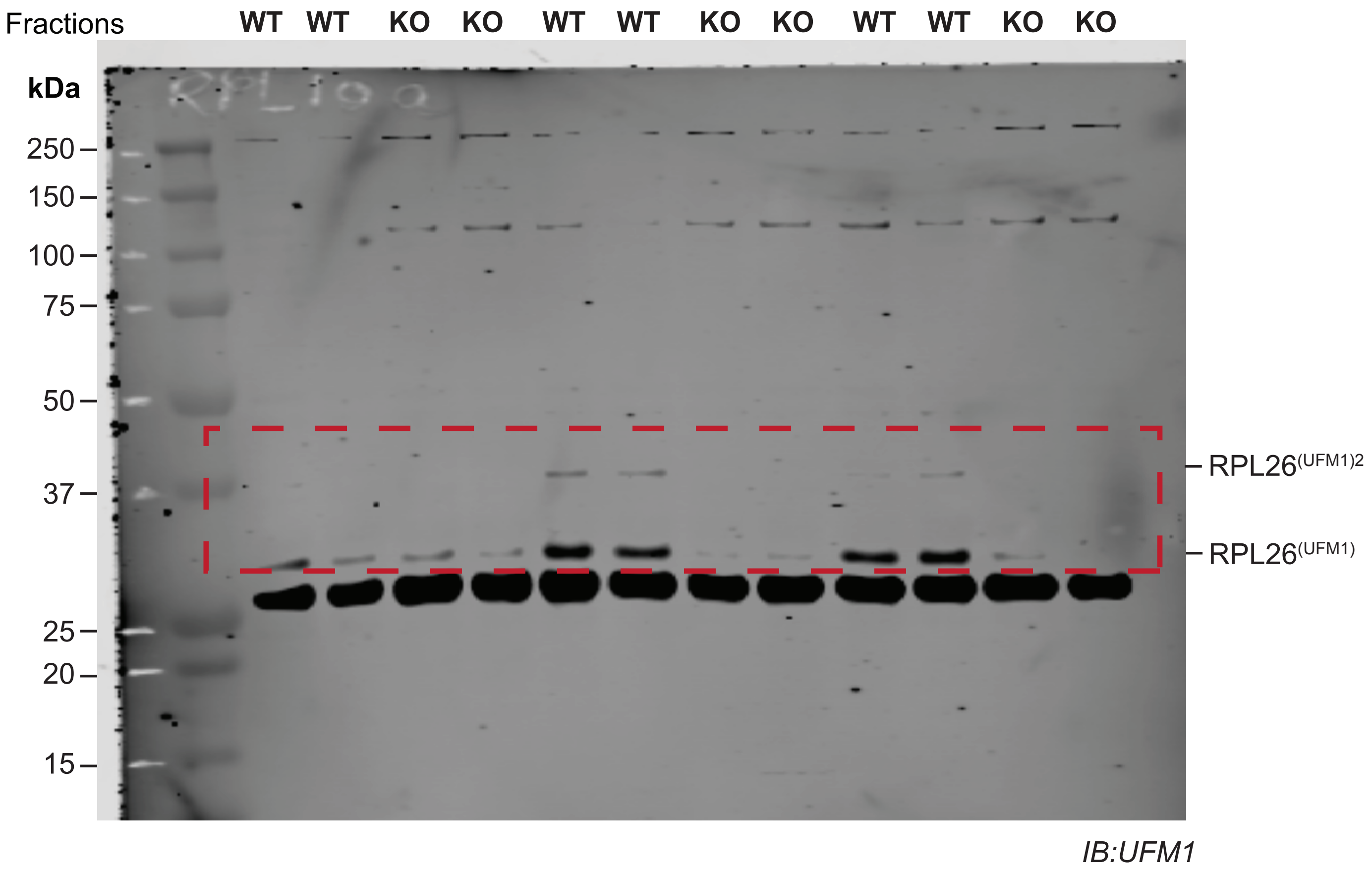
Fractions
WT WT KO KO WT WT KO KO WT WT KO KO
kDa
250
150
100
75
50
37
25
20
15
RPL26$^{(UFM1)2}$
RPL26$^{(UFM1)}$
IB:UFM1

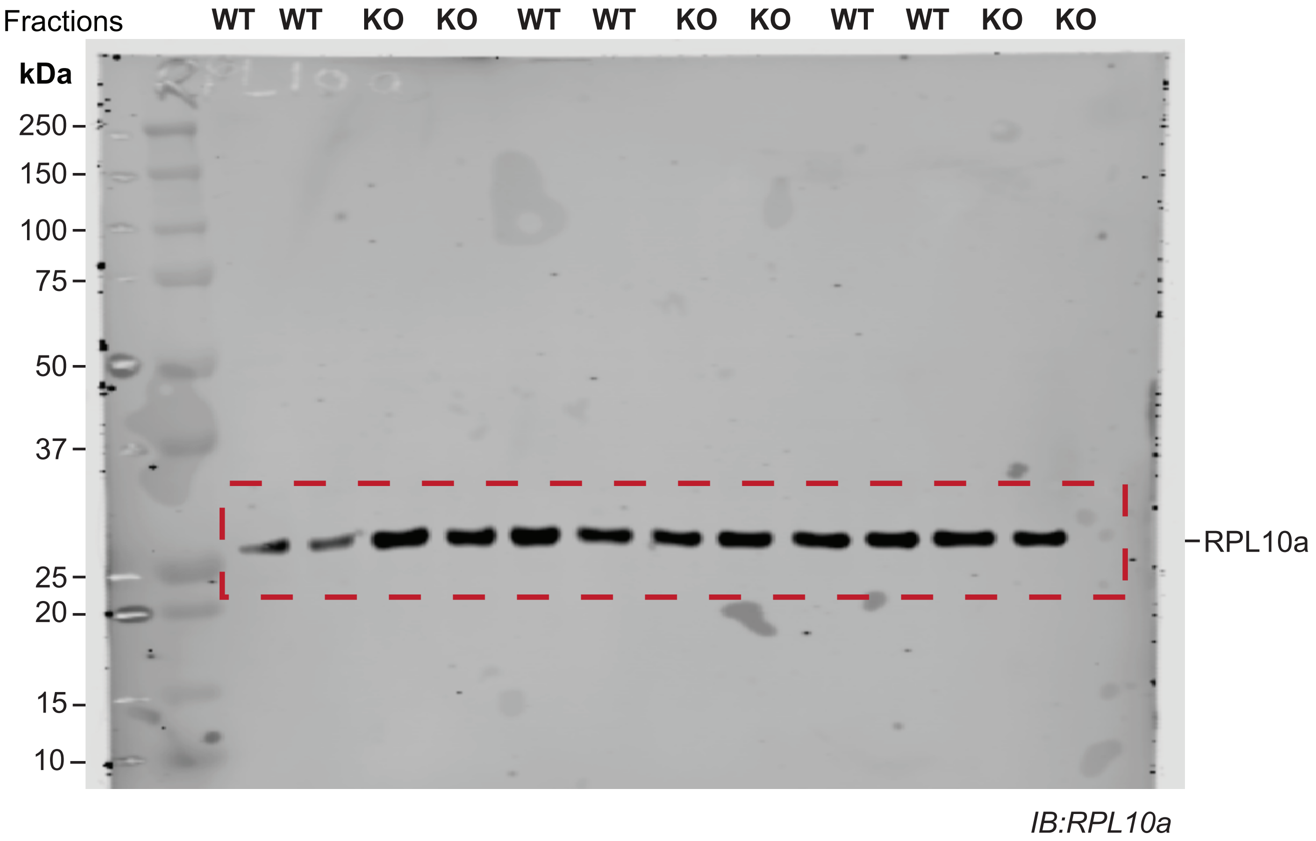
Fractions
WT WT KO KO WT WT KO KO WT WT KO KO
kDa
250
150
100
75
50
37
25
20
15
10
RPL10a
IB:RPL10a
