# Supplementary material for: The UFM1 E3 ligase recognizes and releases 60S ribosomes from ER translocons
Source: Nature. 2024 Feb 21;627(8003):437–44. doi: 10.1038/s41586-024-07093-w (PMC10937380; doi:10.1038/s41586-024-07093-w)
Supplement: Supplementary file 7 — Source Data Extended Data Figs. 1, 3, 5 and 8 and Supplementary Fig. 1. [file 41586_2024_7093_MOESM7_ESM.pdf]

Source Data Figure 2: Uncropped gel scans from Extended Data Figures and Supplementary Figure 1

a, Immunoblots (from Extended Data Figure 1a) comparing UFMylation of 60S and 80S ribosomes *in vitro*. Reaction products were run on a single SDS-PAGE gel and following transfer, the respective membrane was probed for RPL26. The membrane was stripped and re-probed with antibody against UFM1.

b, Immunoblots (from Extended Data Figure 1b) analysing co-migration of UREL components, 60S and 80S ribosomes. Post incubation, the mentioned components were separated on a sucrose gradient and run on two 4-12% SDS-PAGE gels under reducing conditions and transferred onto two different membranes. Membrane 1 was cut into three sections and blotted for UFBP1and RPL26. Membrane 2 was cut into two and blotted for CDK5RAP3 and RPS10.

c, Immunoblots (from Extended Data Figure 1c and 1d) of polysome profile from membrane fraction of WT Flp-In 293 T-REx cells under normal and stalling (anisomycin-treated) conditions. For each condition samples were run on two different SDS-PAGE gels and transferred onto two separate membranes. Membrane 1 and 2 were cut into two sections. The upper section of membrane 1 was probed for UFL1 and the lower section was probed for RPL26 followed by anti-UFBP1. The upper section of membrane 2 was probed for CDK5RAP3 and the lower section was probed for UFM1 followed by anti-RPS10.

e, Immunoblots (from Extended Data Figure 1e) analysing reaction products from an *in vitro* ribosome UFMylation assay. UFMylated ribosomes generated *in vitro* for mass spectrometry analysis were separated on a 4-12% SDS-PAGE gel and immunoblotted for RPL26.

f, Immunoblots (from Extended Figure 1g) analysing the mode of RPL26 poly-UFMylation. *In vitro* ribosome UFMylation was performed in the presence of indicated mutants and run on two 4-12% SDS-PAGE gels, followed by transfer onto two different membranes. Membrane 1 was cut into two and blotted for UFL1 and RPL26. Membrane 2 was cut and blotted for RPL26.

g, Coomassie-stained SDS-PAGE gel (from Extended Data Figure 1i) showing purified UREL, UFC1~UFM1 mimic and 60S ribosomes used in the preparation of samples for visualization by cryo-EM.

h, Coomassie-stained SDS-PAGE gel (from Extended Data Figure 1j) showing *in vitro* reaction for the generation of UFC1-UFM1.

i, Coomassie-stained SDS-PAGE gel (from Extended Data Figure 1k) analysing stability of UFC1-UFM1. Purified UFC1-UFM1 was incubated with UFL1/UFBP1 at 37 °C to

monitor hydrolysis of UFC1-UFM1 conjugate. The reaction was stopped at indicated time points and separated on a 4-12% SDS-PAGE gel followed by Coomassie staining.

j, Immunoblots (from Extended Data Figure 1m) analysing co-migration of UREL/60S/UFC1-UFM1 complexes in the presence of glutaraldehyde. Post sucrose – gradient fractionation, the fractions were run on four different 4-12% SDS-PAGE gels under reducing conditions and transferred onto four different membranes. The membranes were then blotted to analyse co-migration of UREL, 60S, UFC1-UFM1 using indicated antibodies.

k, Immunoblots (from Extended Data Figure 3j) of membrane fraction from HEK293 WT, CDK5RAP3 KO and UFSP2, ODR4 double KO cells untreated or treated with 200nM anisomycin for 60 min. Six different 4-12% SDS-PAGE gels were run and transferred onto membranes for western blotting. Membrane 1 was probed for UFM1, followed by CDK5RAP3. The membrane was stripped and re-probed for ERp72. Membrane 2 was probed for RPL26 by anti-UFSP2 antibody. Membranes 3, 4 and 5 were probed for UFL1, ODR4 and ERp72, respectively. Membrane 6 was probed for UFSP2, followed by UFBP1. Asterisk indicates empty lane.

l, Immunoblots (from Extended Data Figure 5b) analysing *in vitro* ribosome UFMylation in the presence of UFBP1 UFIM mutants. The reaction products were run on a 4-12% SDS-PAGE gel and transferred onto a membrane. The membrane was cut into two sections and immunoblotted for UFL1 and RPL26. The blot shown in the extended data figure is representative of 3 independent experiments.

m, Coomassie-stained SDS-PAGE gel (from Extended Data Figure 5e (top, bottom)) showing SEC fractions of $E3_{mUU}$-ΔUFIM and $E3_{mUU}$-ΔUFIM:UFC1, respectively.

n, Coomassie-stained SDS-PAGE gel (from Extended Data Figure 5e (middle), 5f (bottom)) showing SEC fractions of UFC1 and $E3_{mUU}$-ΔUFIM:UFC1 L32R, respectively.

o, Coomassie-stained SDS-PAGE gel (from Extended Data Figure 5f (top), 5g (bottom)) showing SEC fractions of UFC1 L32R and $E3_{mUU}$-ΔUFIM:UFC1 I40R, respectively.

p, Coomassie-stained SDS-PAGE gel (from Extended Data Figure 5m (bottom)) showing SEC fractions of $E3_{mUU}$-ΔUFIM:UFC1 D50A.

q, Coomassie-stained SDS-PAGE gel (from Extended Data Figure 5m (top), 5h (top)) showing SEC fractions of UFC1 D50A and $E3_{mUU}$(UFL1 I8R)-ΔUFIM, respectively.

r, Coomassie-stained SDS-PAGE gel (from Extended Data Figure 5h (bottom), 5i (top)) showing SEC fractions of $E3_{mUU}$(UFL1 I8R)-ΔUFIM:UFC1 and $E3_{mUU}$(UFL1 L11R)-ΔUFIM, respectively.

s, Coomassie-stained SDS-PAGE gel (from Extended Data Figure 5i (bottom), 5j (top)) showing SEC fractions of $E3_{mUU}$(UFL1 L11R)-ΔUFIM:UFC1 and $E3_{mUU}$(UFL1 F15R)-ΔUFIM, respectively.

t, Coomassie-stained SDS-PAGE gel (from Extended Data Figure 5j (bottom), 5k (top)) showing SEC fractions of $E3_{mUU}$(UFL1 F15R)-ΔUFIM:UFC1 and $E3_{mUU}$(UFL1 Q19R)-ΔUFIM, respectively.

u, Coomassie-stained SDS-PAGE gel (from Extended Data Figure 5k (bottom), 5n (top)) showing SEC fractions of $E3_{mUU}$(UFL1 Q19R)-ΔUFIM:UFC1 and $E3_{mUU}$(UFBP1 R265A)-ΔUFIM, respectively.

v, Coomassie-stained SDS-PAGE gel (from Extended Data Figure 5n (bottom), 5g (top)) showing SEC fractions of $E3_{mUU}$(UFBP1 R265A)-ΔUFIM:UFC1 and UFC1 I40R, respectively.

w, Coomassie-stained SDS-PAGE gel under non-reducing conditions (from Extended Data Figure 5l) showing aminolysis of UFM1 from UFC1~UFM1 in the presence of $E3_{mUU}$-ΔUFIM mutants.

x, Coomassie-stained SDS-PAGE gel (from Extended Data Figure 7e) showing SEC fractions of UFL1-Δα1/UFBP1, $E3_{mUU}$, CDK5RAP3 and UFC1-UFM1.

y, Coomassie-stained SDS-PAGE gel (from Extended Data Figure 7f) showing SEC fractions of $E3_{mUU}$:UFC1-UFM1, $E3_{mUU}$:CDK5RAP3 and $E3_{mUU}$:CDK5RAP3/UFC1-UFM1.

z, Immunoblots from (Extended Data Figure 8g) of sucrose density fractions after *in vitro* UFMylation of membrane associated 60S ribosome-SEC61 complexes isolated from CDK5RAP3 KO cells in the presence and absence of ATP. Two different gels were run and transferred onto separate membranes. Membrane 1 was probed for RPL26 followed by anti-UFL1. Membrane 2 was cut into two sections. The lower section was probed for SEC61β and the upper section was probed for UFM1 followed by anti-UFBP1.

aa, Immunoblots (from Extended Data Figure 8h) of *in vitro* 60S-SEC61 UFMylation and translocon dissociation assay performed as in Extended Data Figure 8g using UREL with the UFBP1 UFIM mutant F196A. Two different gels and respective membranes were run. Membrane 1 was probed for RPL26. Membrane 2 was cut into two sections. The lower section was probed for SEC61β and the upper section was probed for UFM1.

ab, Coomassie-stained SDS-PAGE gel (from Supplementary Data Figure 3a) analysing formation of UREL-ribosome complexes in the presence and absence of the crosslinker DSBU.

a

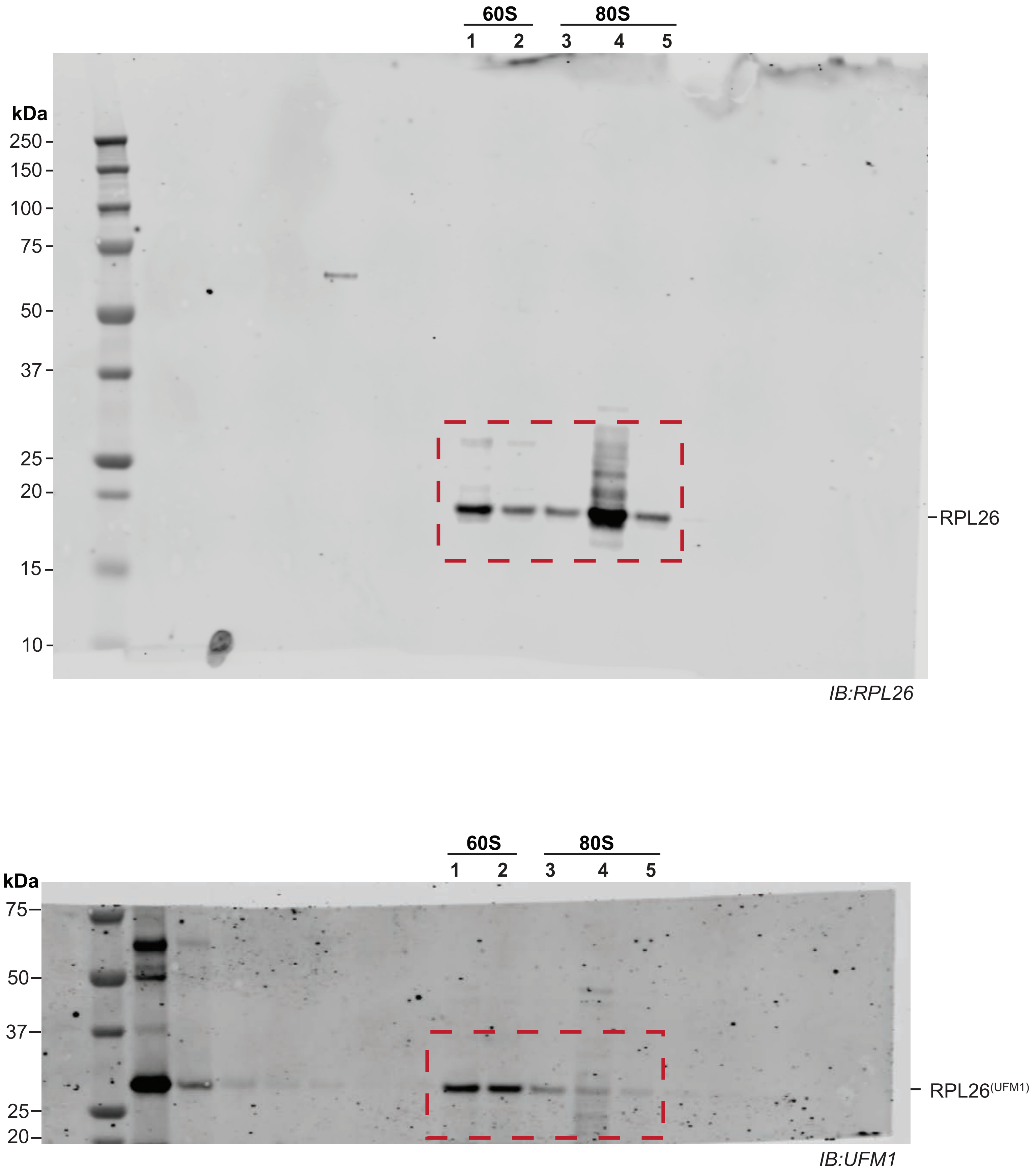
60S
80S
1
2
3
4
5
kDa
250
150
100
75
50
37
25
20
15
10
RPL26
IB:RPL26
60S
80S
1
2
3
4
5
kDa
75
50
37
25
20
RPL26(UFM1)
IB:UFM1

b

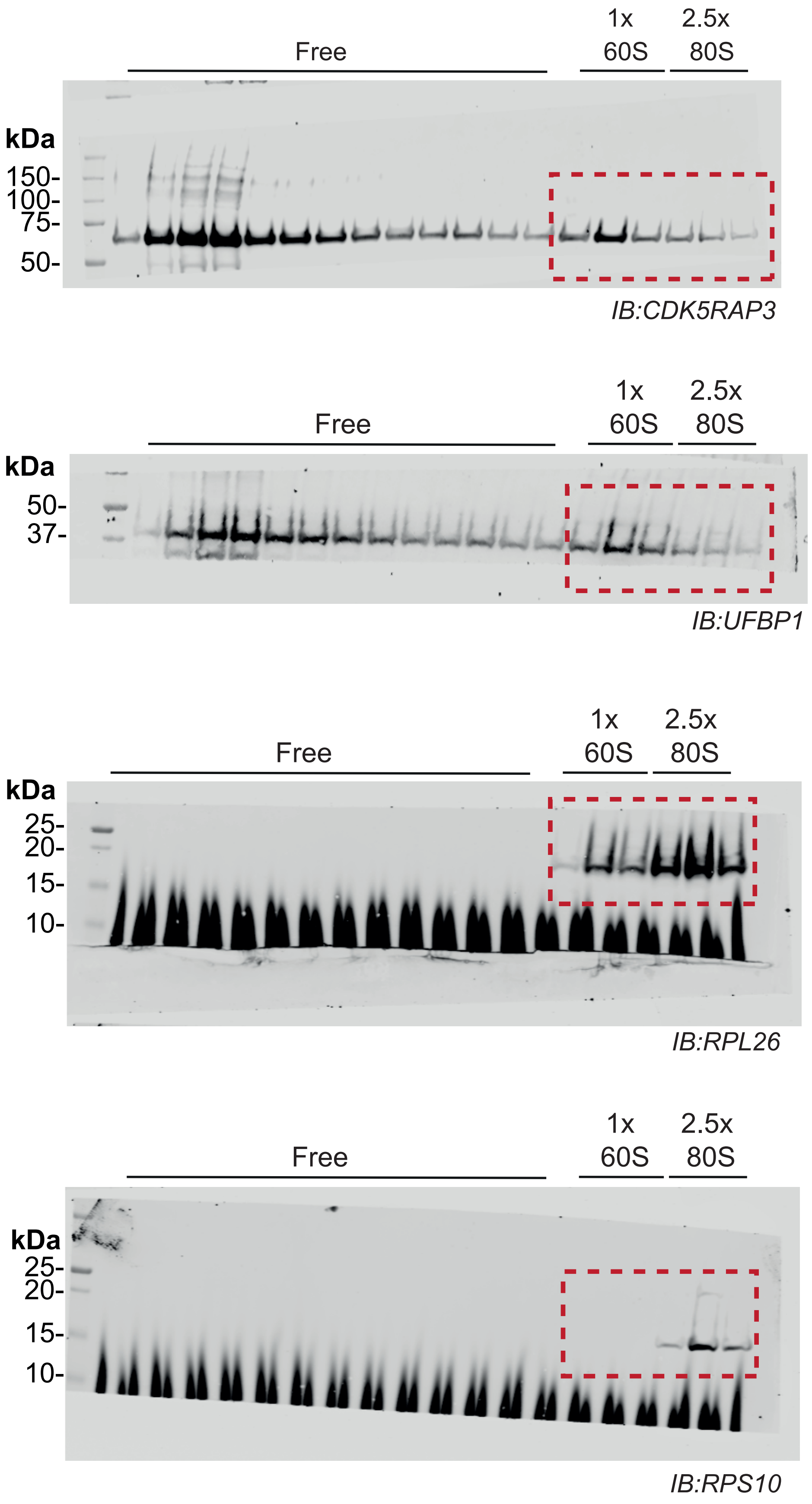
1x
60S
2.5x
80S
Free
kDa
150-
100-
75-
50-
IB:CDK5RAP3
1x
60S
2.5x
80S
Free
kDa
50-
37-
IB:UFBP1
1x
60S
2.5x
80S
Free
kDa
25-
20-
15-
10-
IB:RPL26
1x
60S
2.5x
80S
Free
kDa
25-
20-
15-
10-
IB:RPS10

c

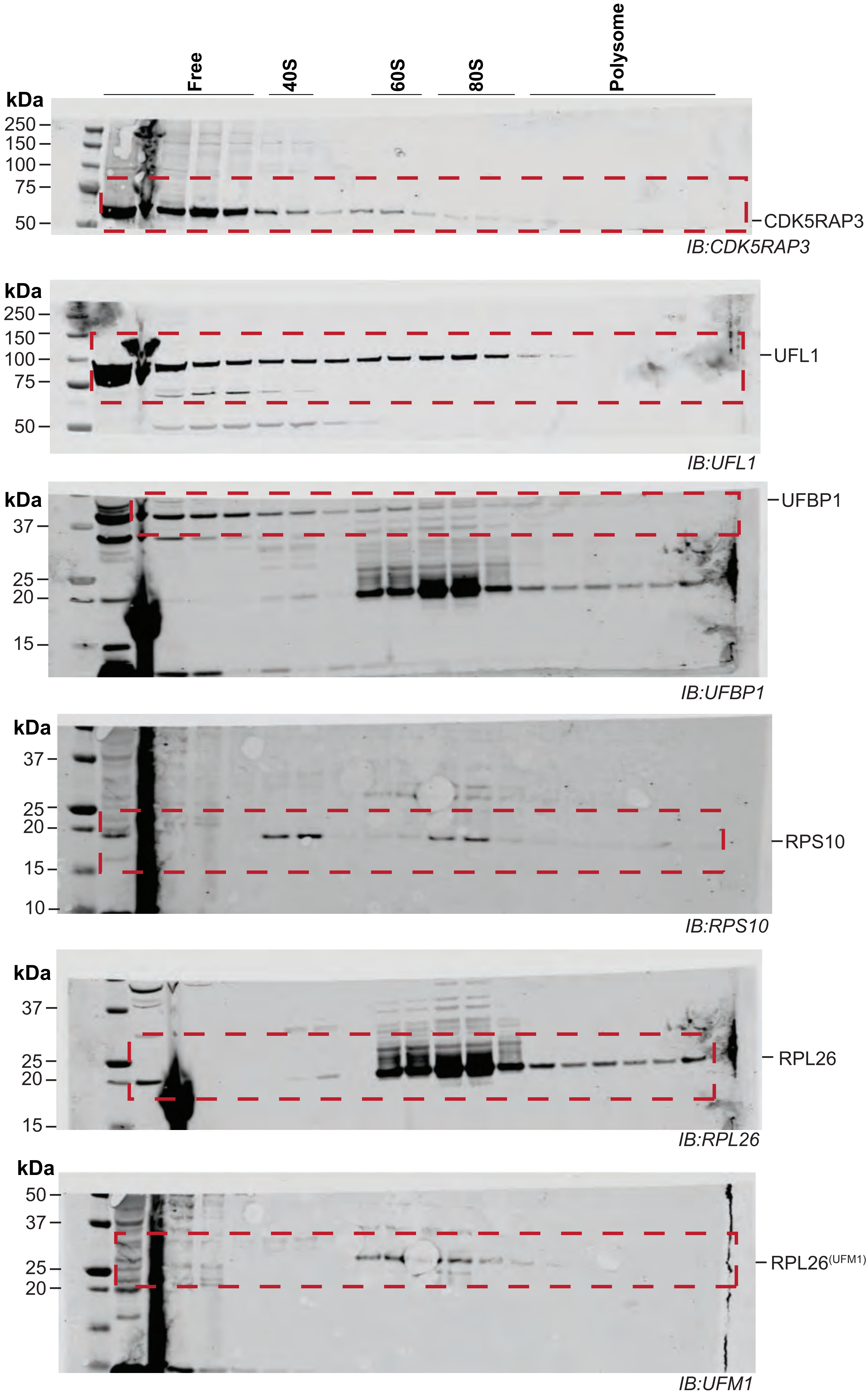
Free
40S
60S
80S
Polysome
kDa
250
150
100
75
50
CDK5RAP3
IB:CDK5RAP3
kDa
250
150
100
75
50
UFL1
IB:UFL1
kDa
37
25
20
15
UFBP1
IB:UFBP1
kDa
37
25
20
15
10
RPS10
IB:RPS10
kDa
37
25
20
15
RPL26
IB:RPL26
kDa
50
37
25
20
RPL26(UFM1)
IB:UFM1

d

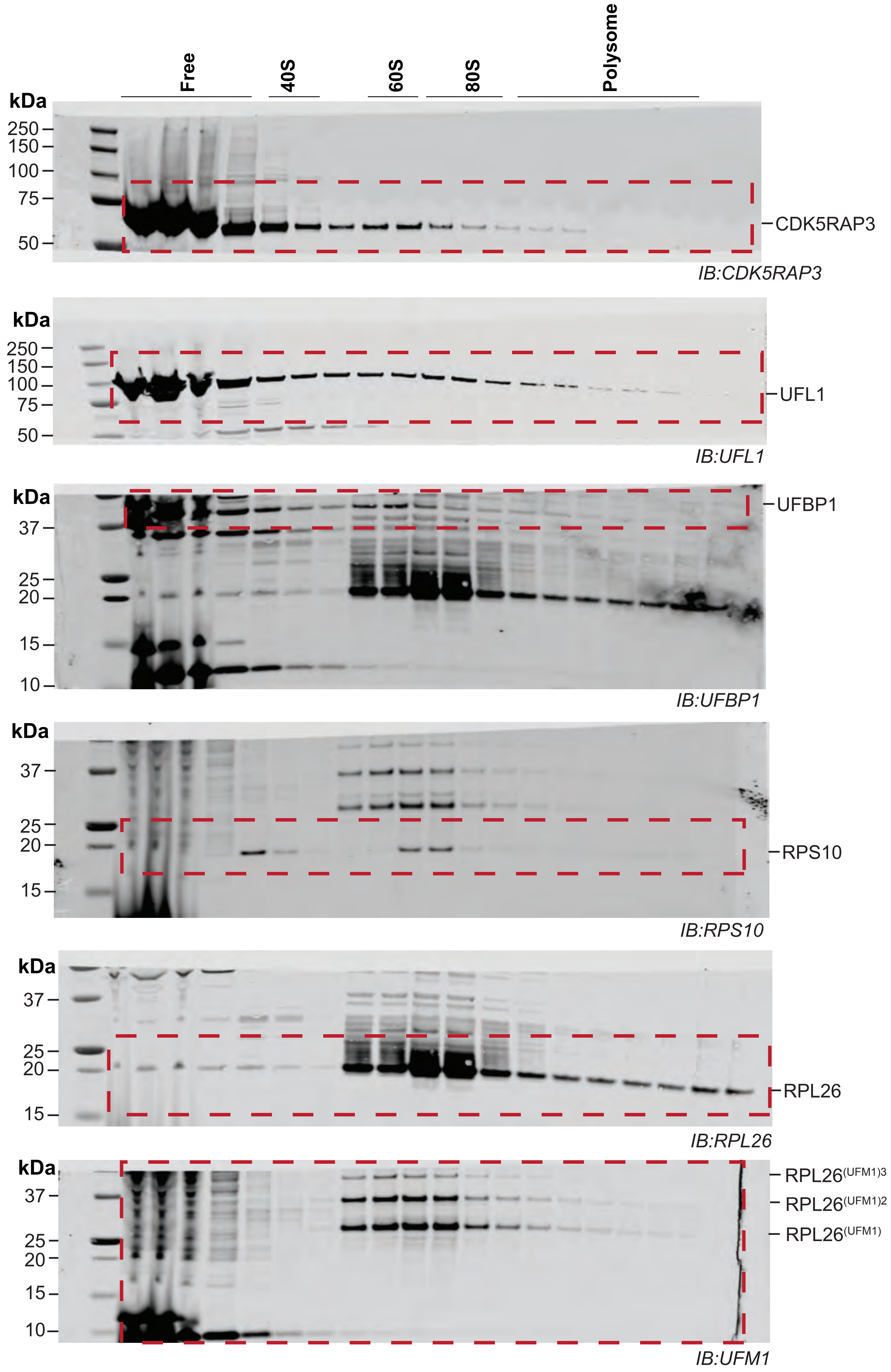

Free
40S
60S
80S
Polysome
kDa
250
150
100
75
50
CDK5RAP3
IB:CDK5RAP3
kDa
250
150
100
75
50
UFL1
IB:UFL1
kDa
37
25
20
15
10
UFBP1
IB:UFBP1
kDa
37
25
20
15
RPS10
IB:RPS10
kDa
37
25
20
15
RPL26
IB:RPL26
kDa
37
25
20
15
10
RPL26(UFM1)3
RPL26(UFM1)2
RPL26(UFM1)
IB:UFM1

e

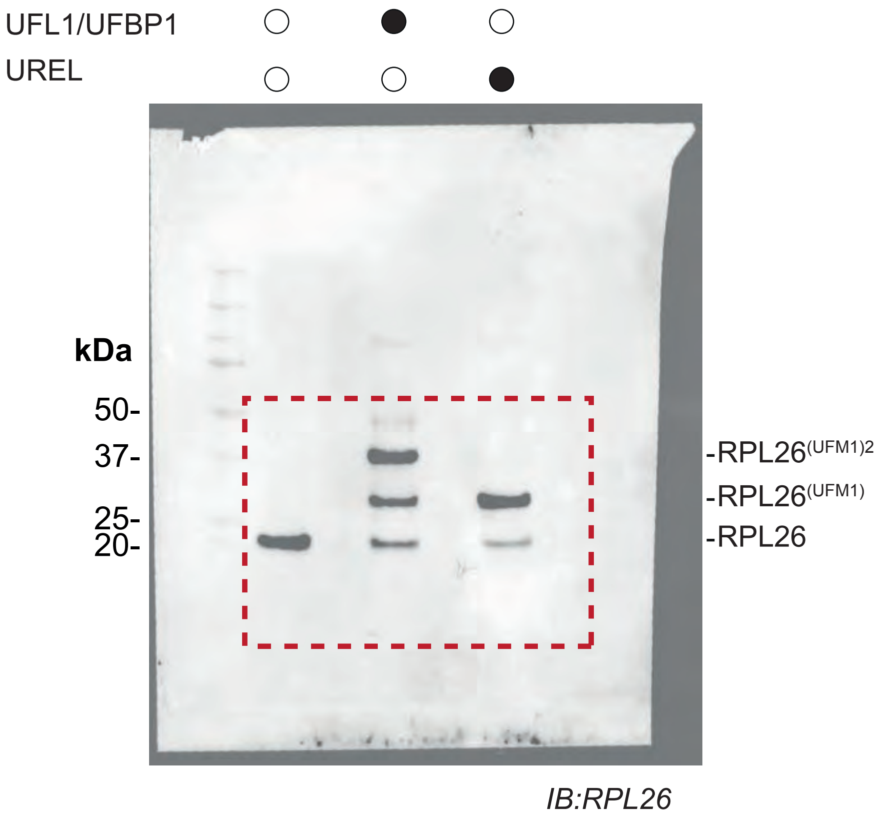

UFL1/UFBP1
UREL
kDa
50-
37-
25-
20-
-RPL26(UFM1)2
-RPL26(UFM1)
-RPL26
IB:RPL26

f

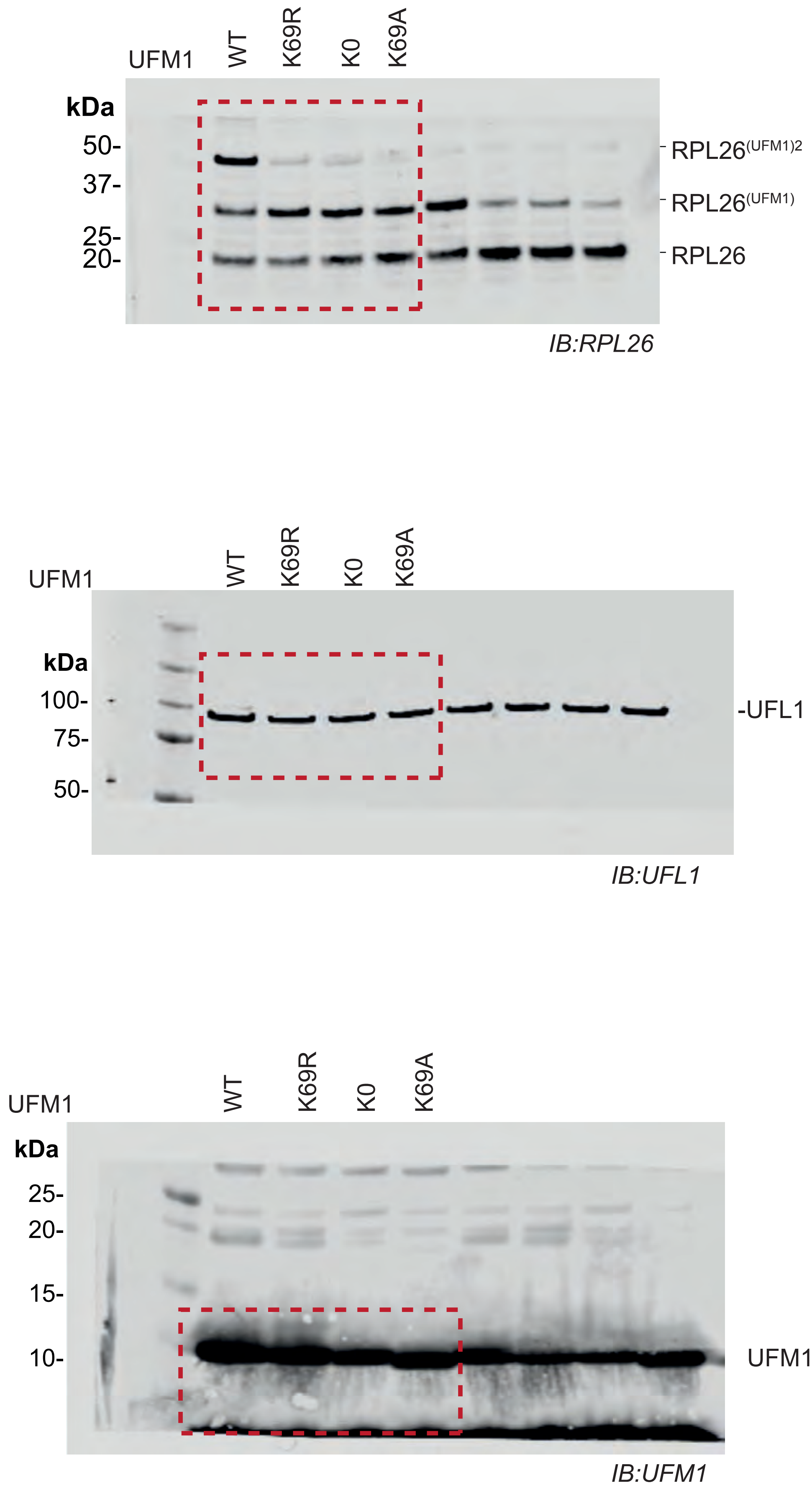
UFM1
WT
K69R
K0
K69A
kDa
50-
37-
25-
20-
RPL26(UFM1)2
RPL26(UFM1)
RPL26
IB:RPL26
UFM1
WT
K69R
K0
K69A
kDa
100-
75-
50-
-UFL1
IB:UFL1
UFM1
WT
K69R
K0
K69A
kDa
25-
20-
15-
10-
UFM1
IB:UFM1

g

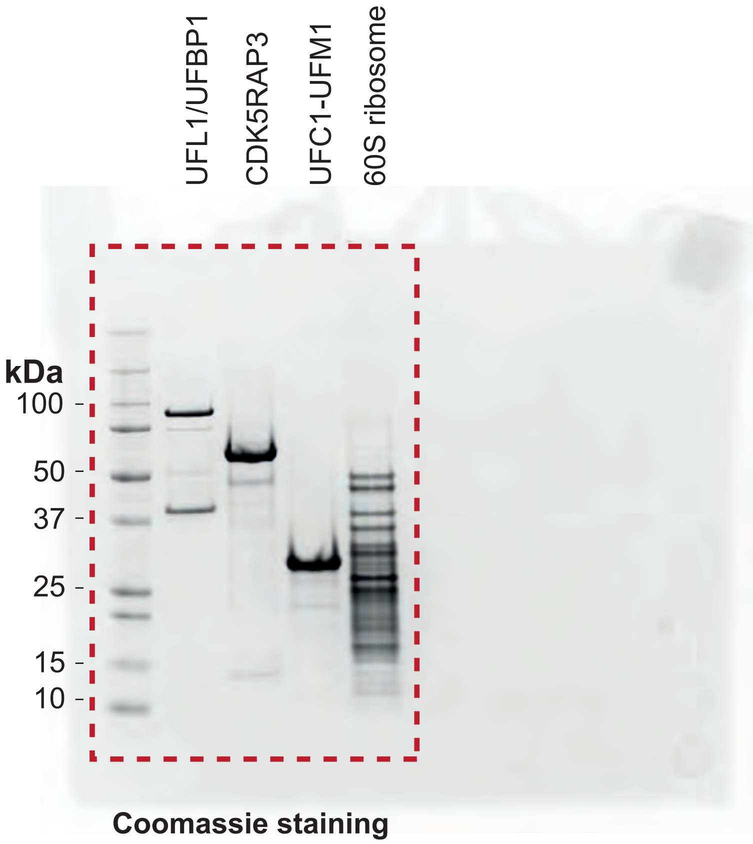

Coomassie staining

h

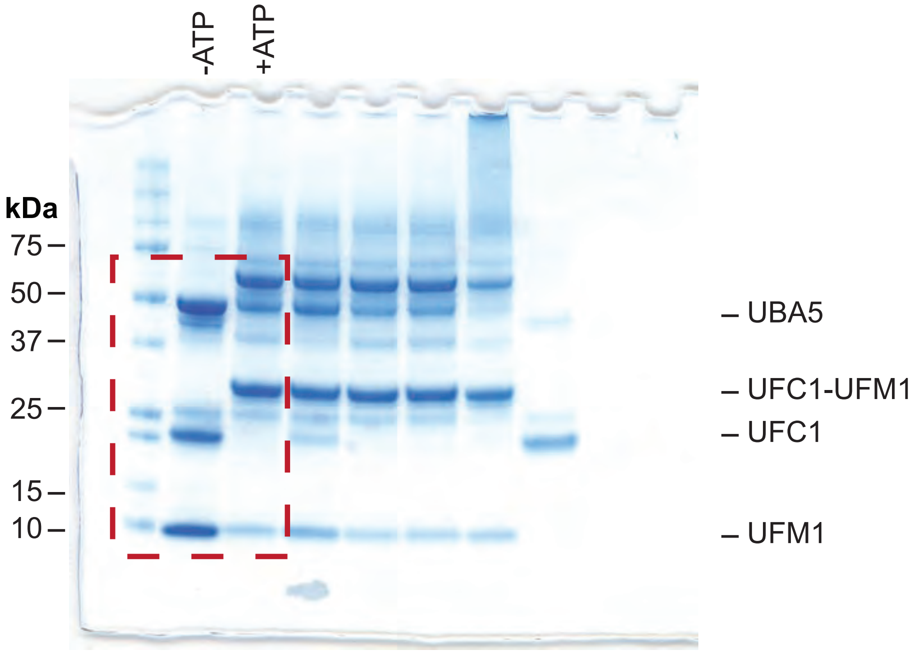

i

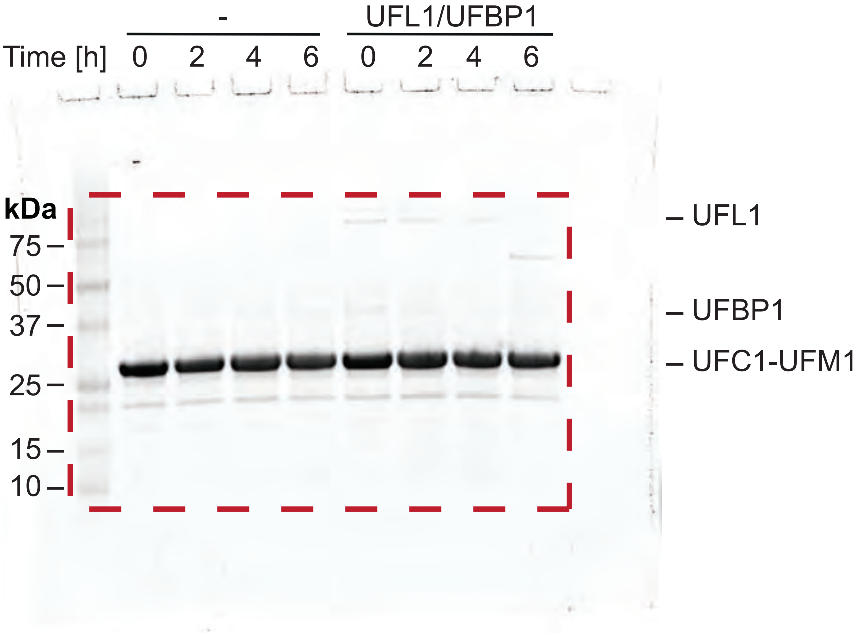

j

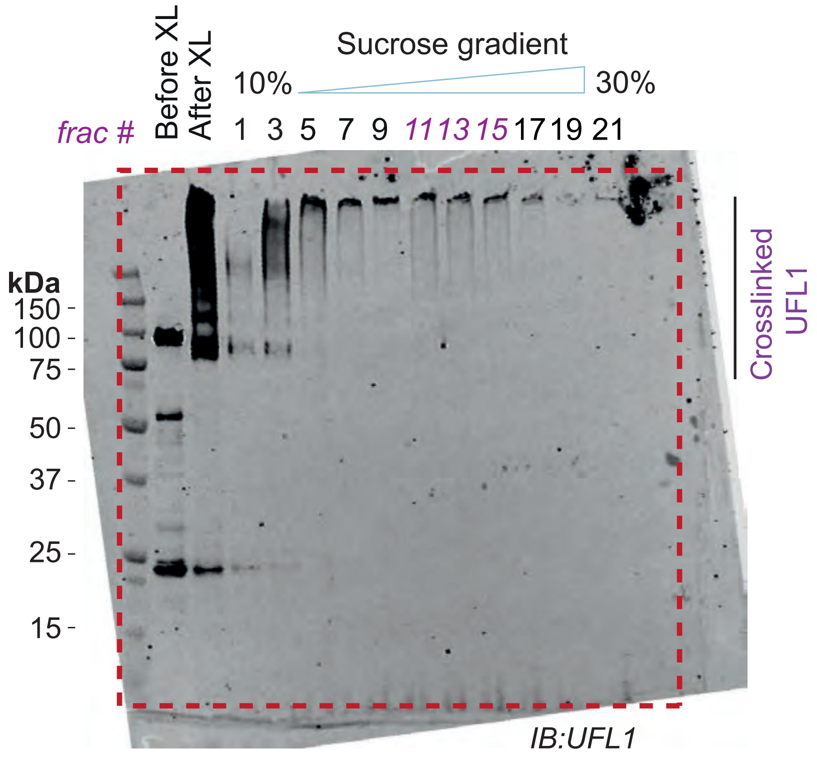

Sucrose gradient
10%
30%
frac #
Before XL
After XL
1 3 5 7 9 11 13 15 17 19 21
kDa
150
100
75
50
37
25
15
Crosslinked UFL1
IB:UFL1

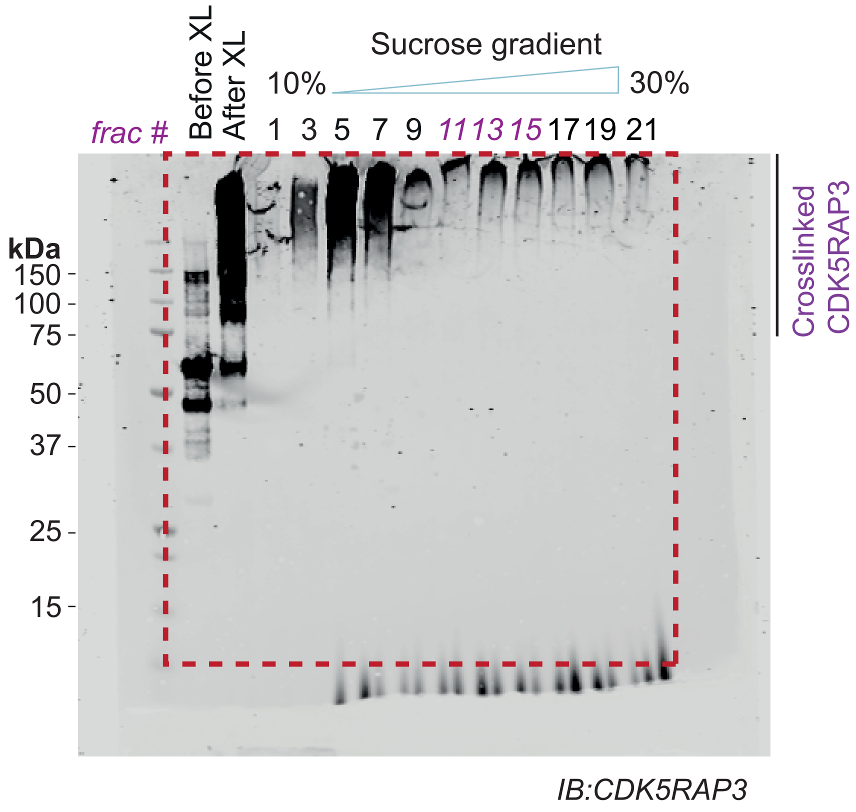

Sucrose gradient
10%
30%
frac #
Before XL
After XL
1 3 5 7 9 11 13 15 17 19 21
kDa
150
100
75
50
37
25
15
Crosslinked CDK5RAP3
IB:CDK5RAP3

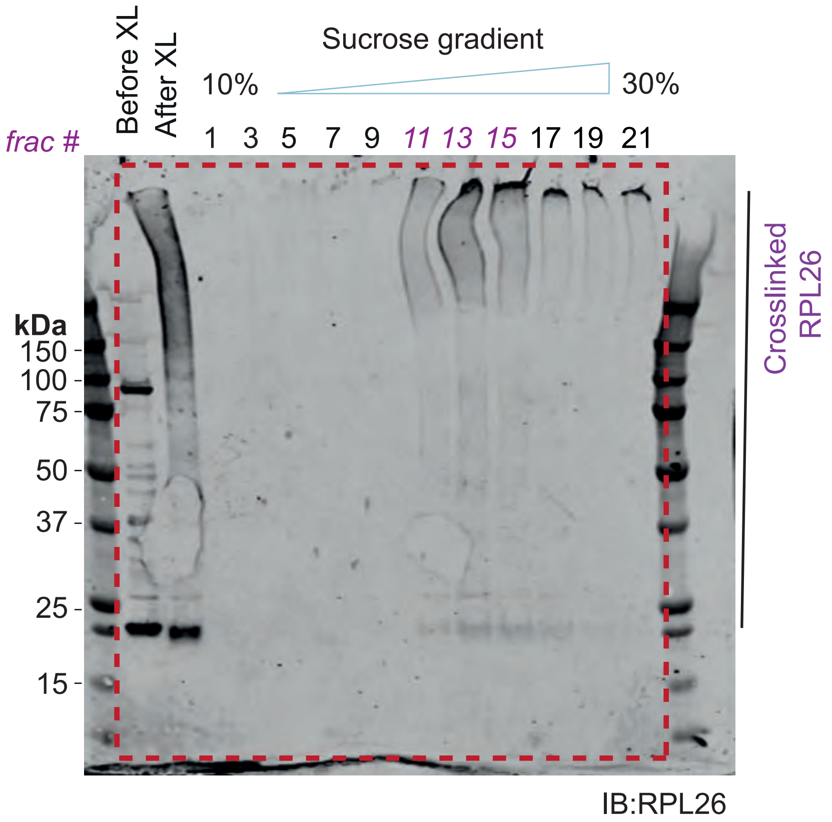
Before XL
After XL
Sucrose gradient
10%
30%
frac #
1 3 5 7 9 11 13 15 17 19 21
kDa
150
100
75
50
37
25
15
Crosslinked RPL26
IB:RPL26

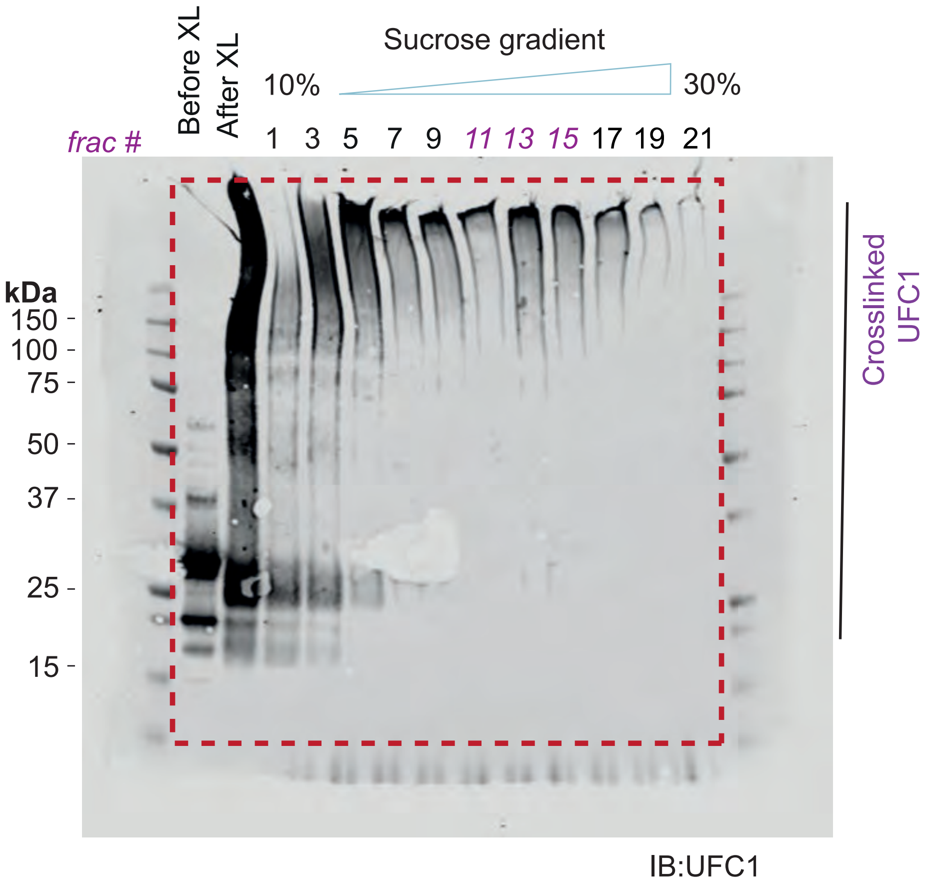
Before XL
After XL
Sucrose gradient
10%
30%
frac #
1 3 5 7 9 11 13 15 17 19 21
kDa
150
100
75
50
37
25
15
Crosslinked UFC1
IB:UFC1

k

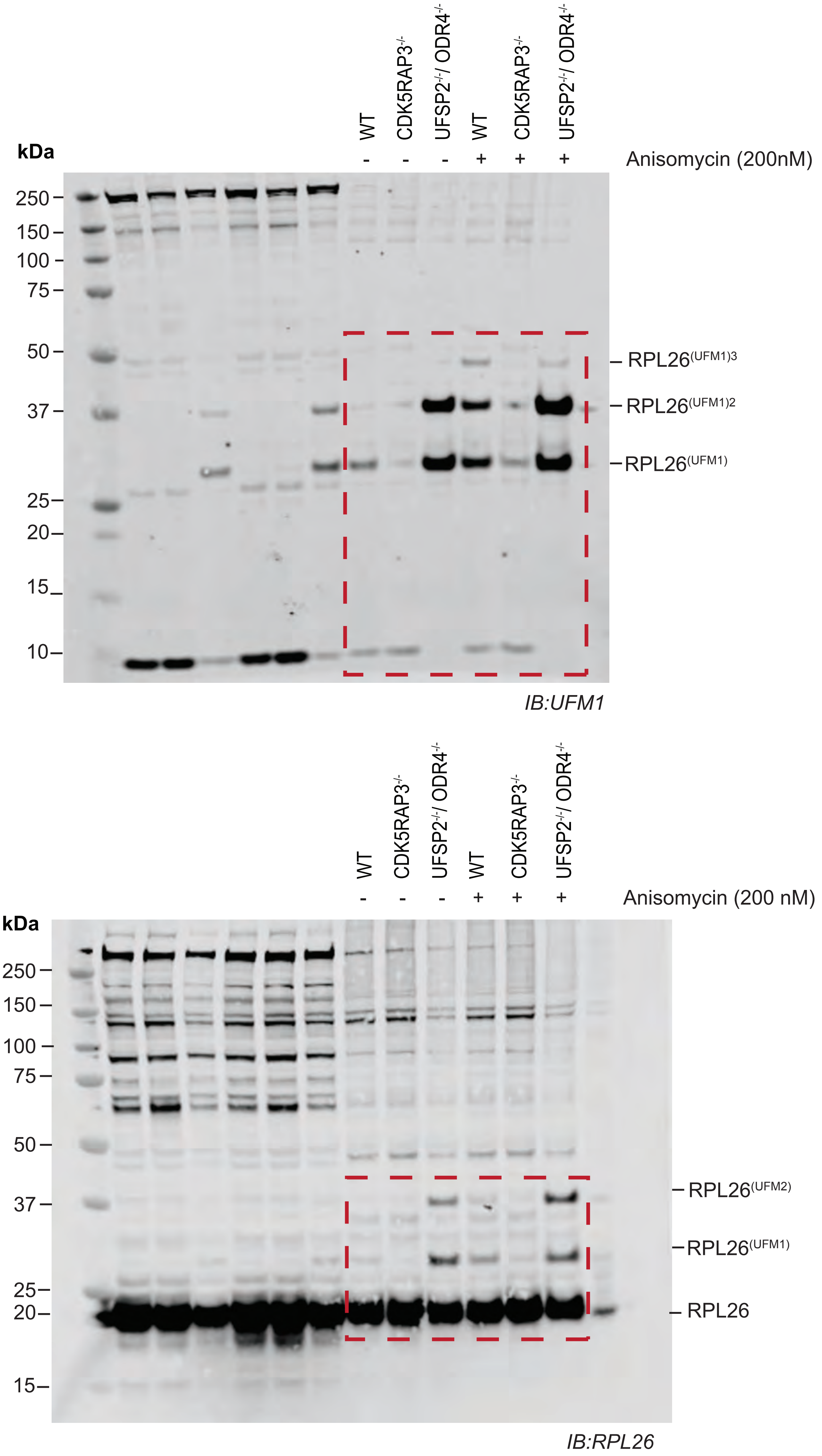
WT
CDK5RAP3-/-
UFSP2-/-/ ODR4-/-
WT
CDK5RAP3-/-
UFSP2-/-/ ODR4-/-
- - - + + +
Anisomycin (200nM)
kDa
250
150
100
75
50
37
25
20
15
10
RPL26(UFM1)3
RPL26(UFM1)2
RPL26(UFM1)
IB:UFM1
WT
CDK5RAP3-/-
UFSP2-/-/ ODR4-/-
WT
CDK5RAP3-/-
UFSP2-/-/ ODR4-/-
- - - + + +
Anisomycin (200 nM)
kDa
250
150
100
75
50
37
25
20
15
RPL26(UFM2)
RPL26(UFM1)
RPL26
IB:RPL26

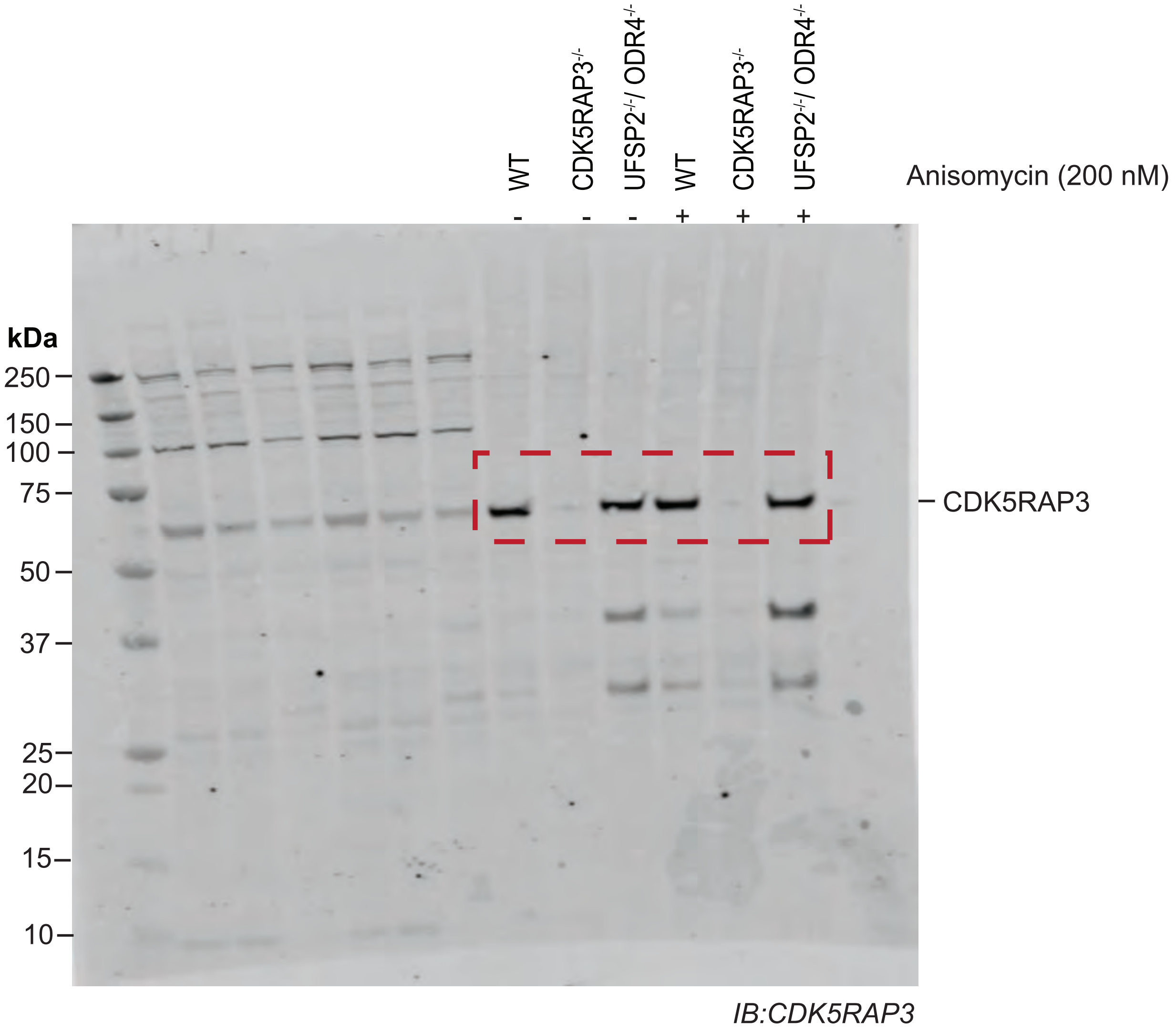
WT
CDK5RAP3-/-
UFSP2-/-/ ODR4-/-
WT
CDK5RAP3-/-
UFSP2-/-/ ODR4-/-
Anisomycin (200 nM)
- - - + + +
kDa
250
150
100
75
50
37
25
20
15
10
CDK5RAP3
IB:CDK5RAP3

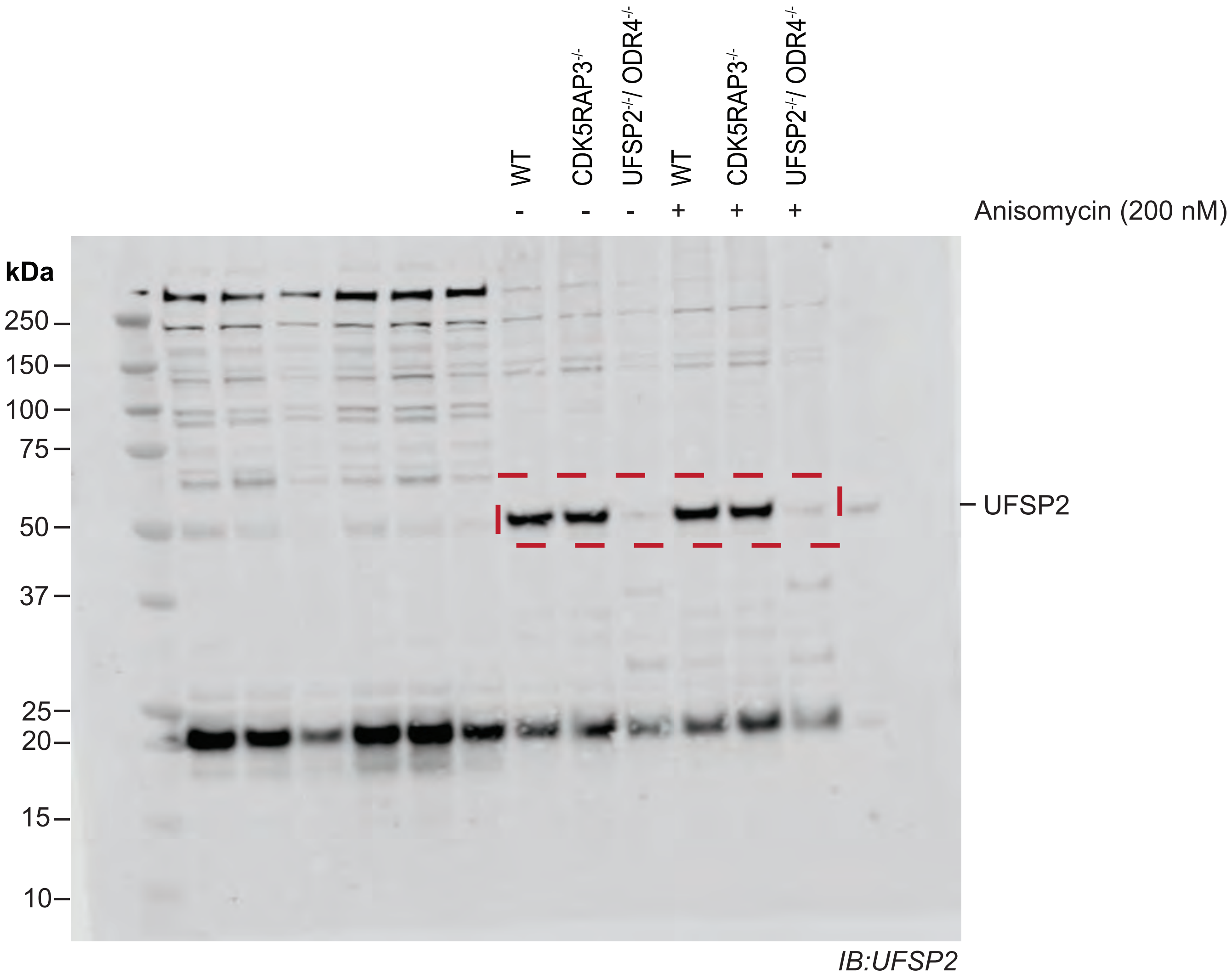
WT
CDK5RAP3-/-
UFSP2-/-/ ODR4-/-
WT
CDK5RAP3-/-
UFSP2-/-/ ODR4-/-
Anisomycin (200 nM)
- - - + + +
kDa
250
150
100
75
50
37
25
20
15
10
UFSP2
IB:UFSP2

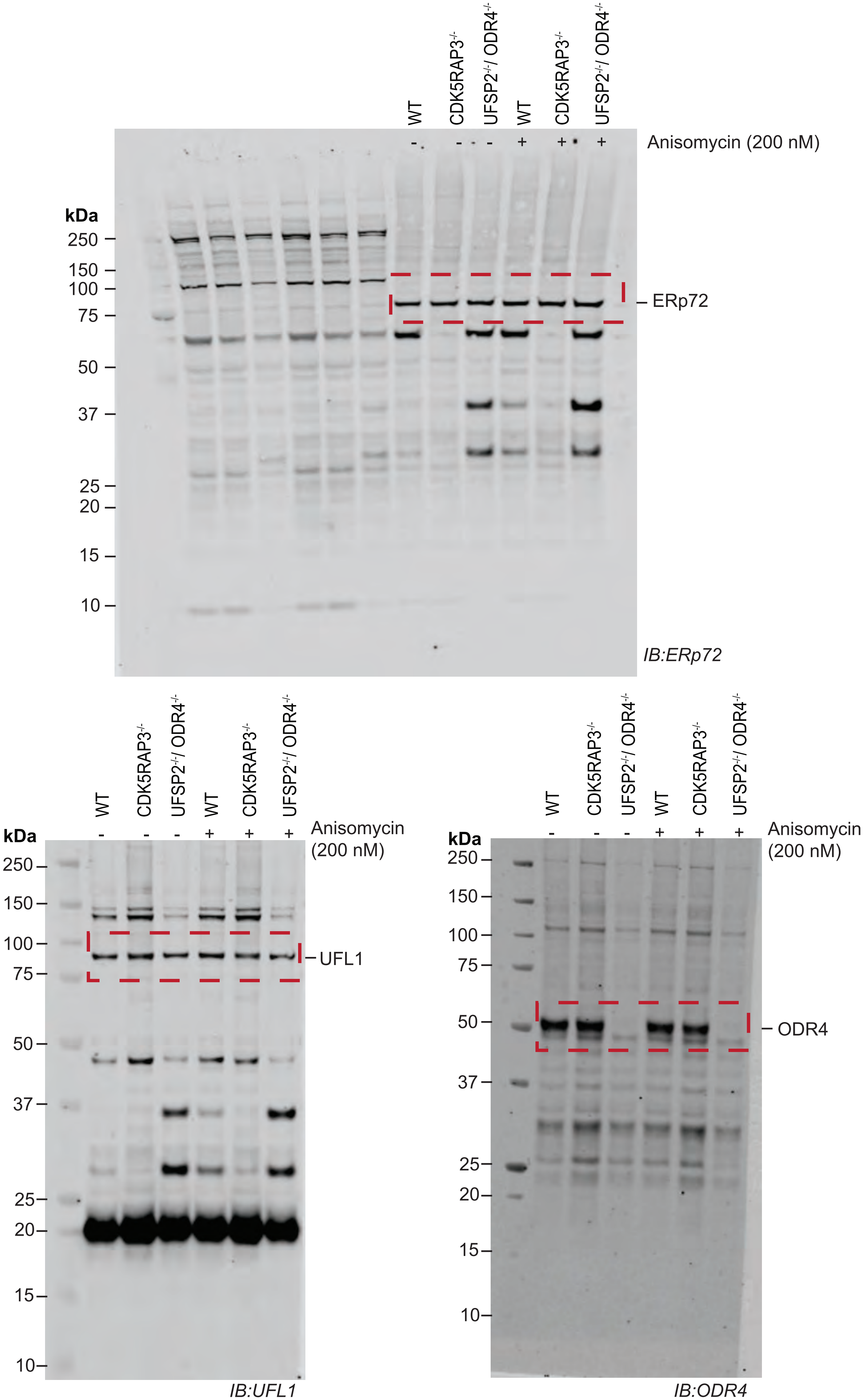
WT
CDK5RAP3-/-
UFSP2-/-/ ODR4-/-
WT
CDK5RAP3-/-
UFSP2-/-/ ODR4-/-
- - - + + +
Anisomycin (200 nM)
kDa
250
150
100
75
50
37
25
20
15
10
ERp72
IB:ERp72
WT
CDK5RAP3-/-
UFSP2-/-/ ODR4-/-
WT
CDK5RAP3-/-
UFSP2-/-/ ODR4-/-
- - - + + +
Anisomycin (200 nM)
kDa
250
150
100
75
50
37
25
20
15
10
UFL1
IB:UFL1
WT
CDK5RAP3-/-
UFSP2-/-/ ODR4-/-
WT
CDK5RAP3-/-
UFSP2-/-/ ODR4-/-
- - - + + +
Anisomycin (200 nM)
kDa
250
150
100
75
50
37
25
20
15
10
ODR4
IB:ODR4

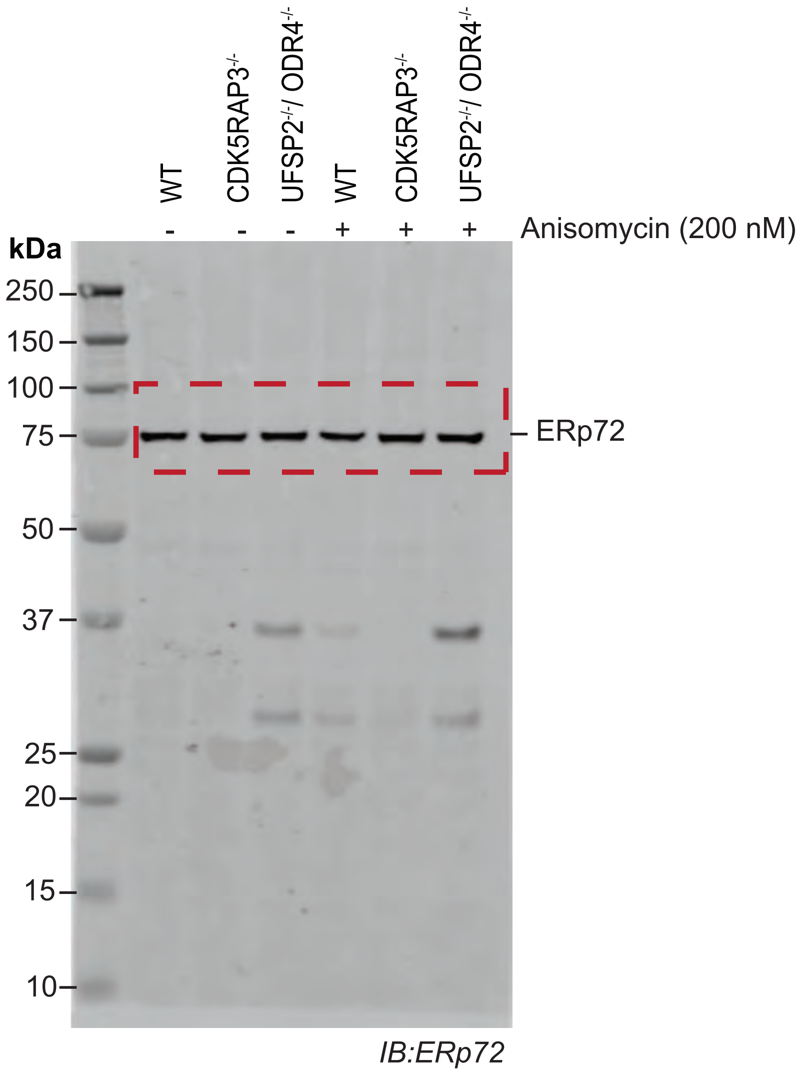
WT
CDK5RAP3-/-
UFSP2-/-/ ODR4-/-
WT
CDK5RAP3-/-
UFSP2-/-/ ODR4-/-
- - - + + +
Anisomycin (200 nM)
kDa
250
150
100
75
50
37
25
20
15
10
ERp72
IB:ERp72

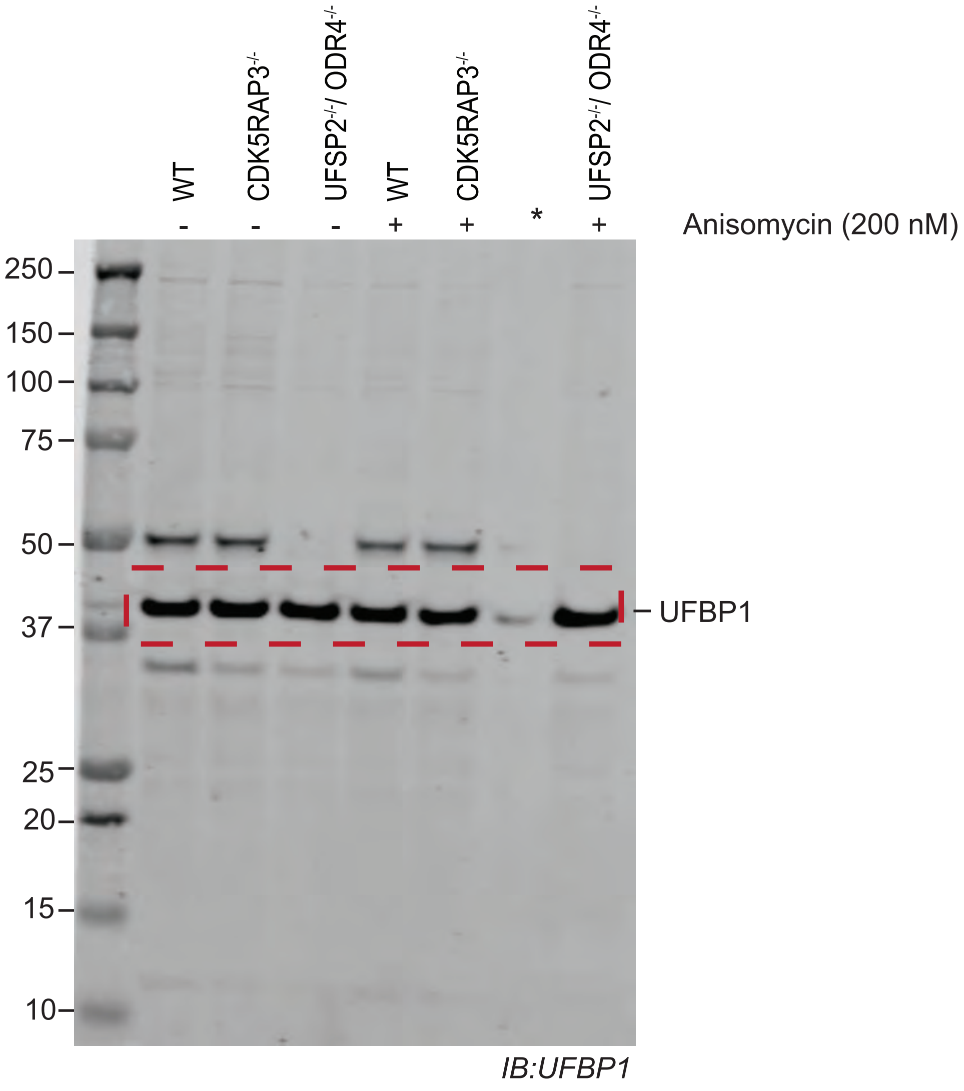
WT
CDK5RAP3-/-
UFSP2-/-/ ODR4-/-
WT
CDK5RAP3-/-
UFSP2-/-/ ODR4-/-
- - - + + * +
Anisomycin (200 nM)
250
150
100
75
50
37
25
20
15
10
UFBP1
IB:UFBP1

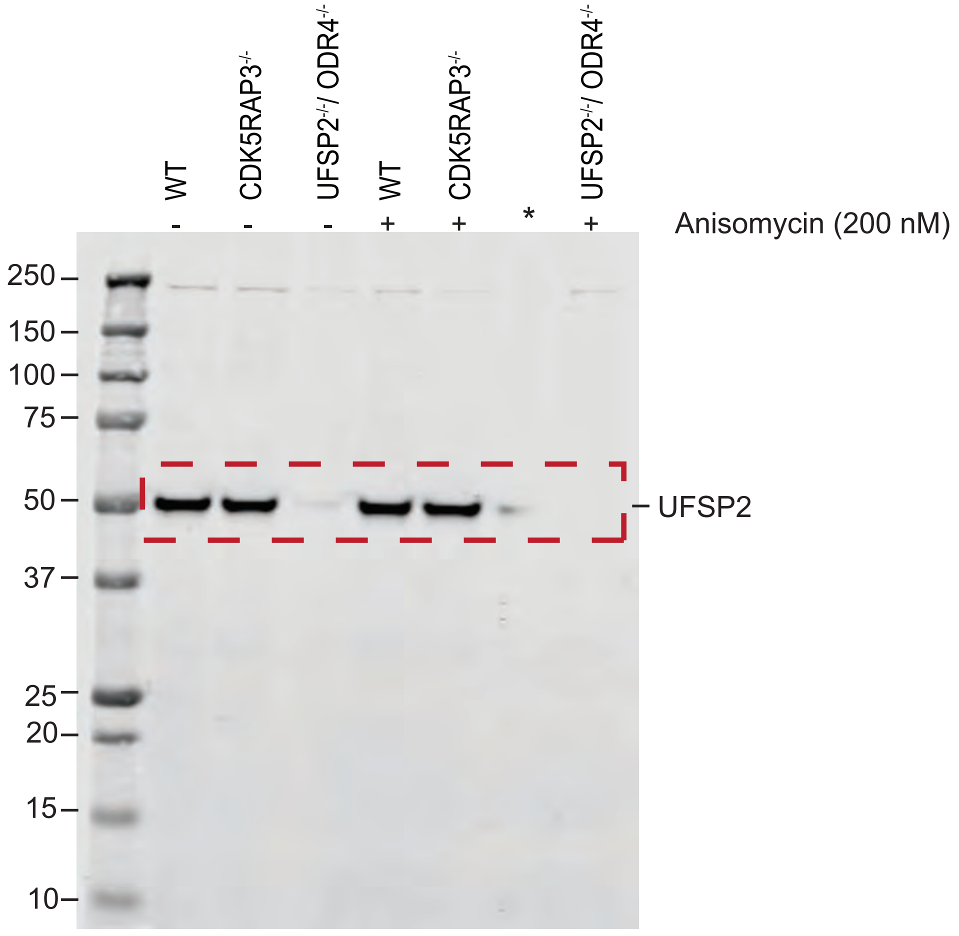

IB:UFSP2

I

Representative blot shown in the manuscript

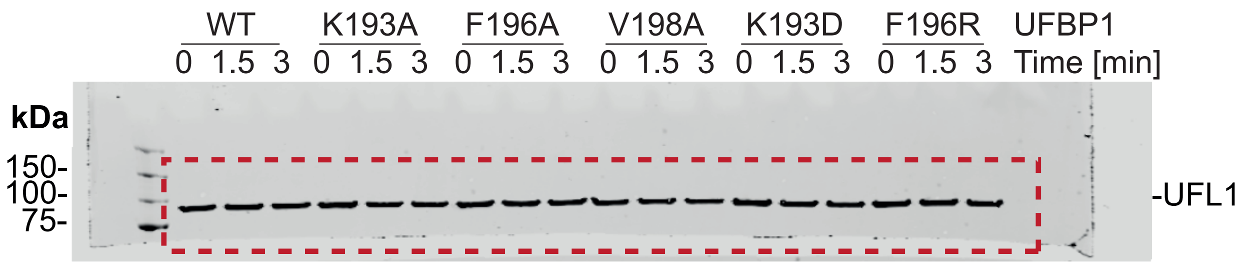

IB:UFL1

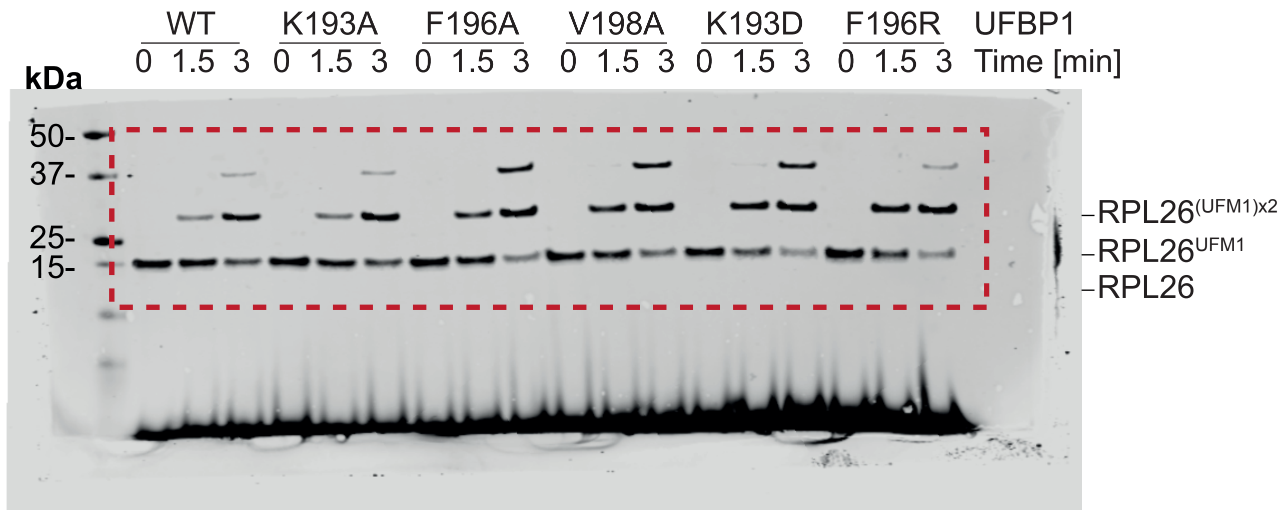

IB:RPL26

m

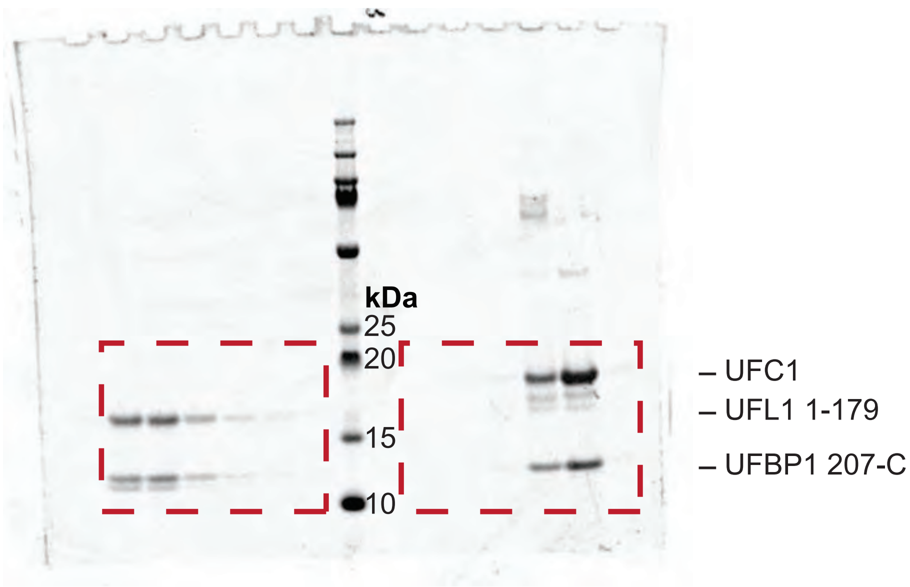
kDa
25
20
15
10
– UFC1
– UFL1 1-179
– UFBP1 207-C

n

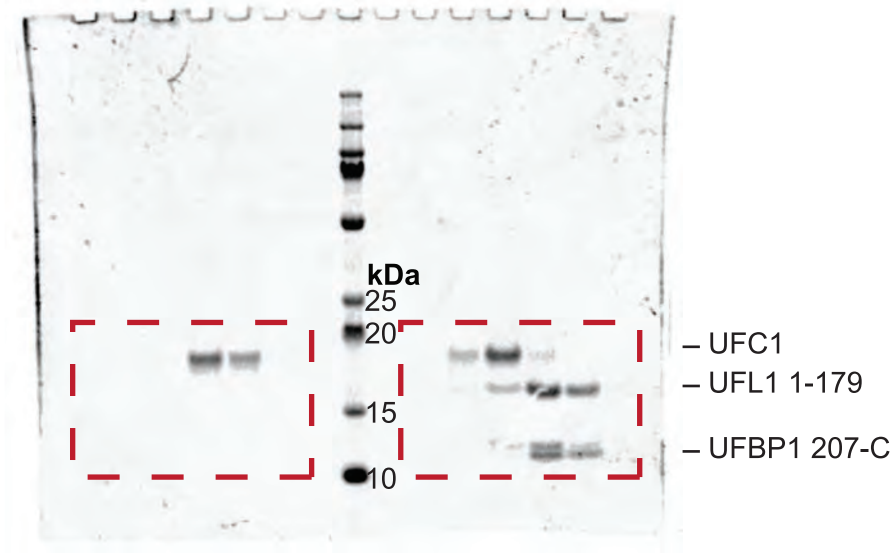
kDa
25
20
15
10
– UFC1
– UFL1 1-179
– UFBP1 207-C

o

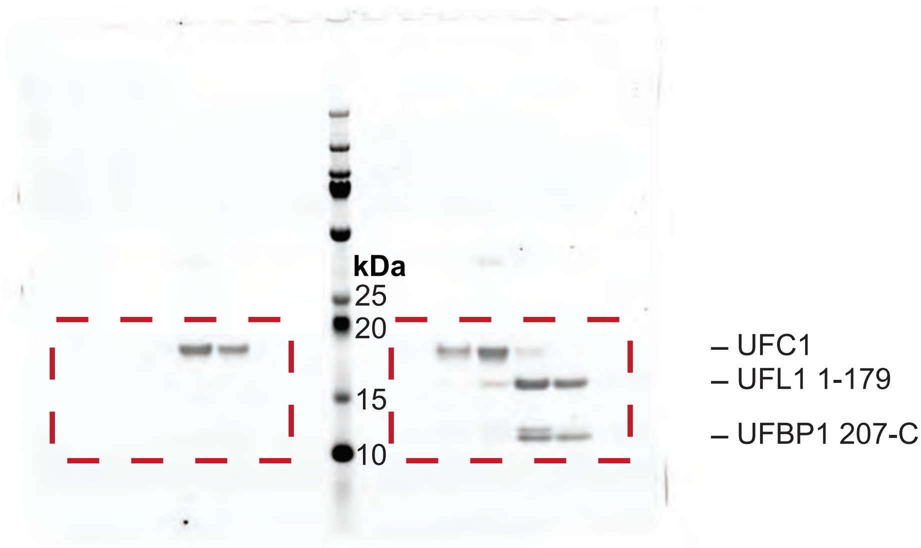
kDa
25
20
15
10
– UFC1
– UFL1 1-179
– UFBP1 207-C

s

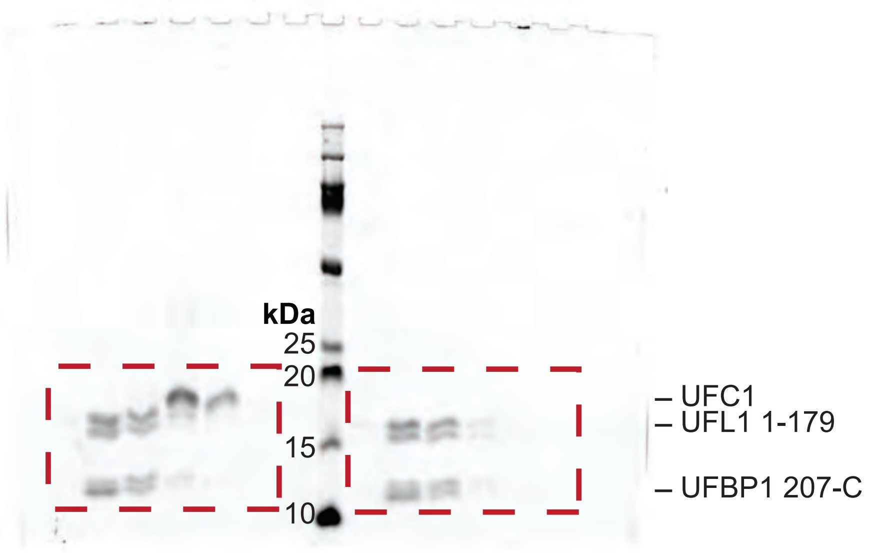
kDa
25
20
15
10
UFC1
UFL1 1-179
UFBP1 207-C

t

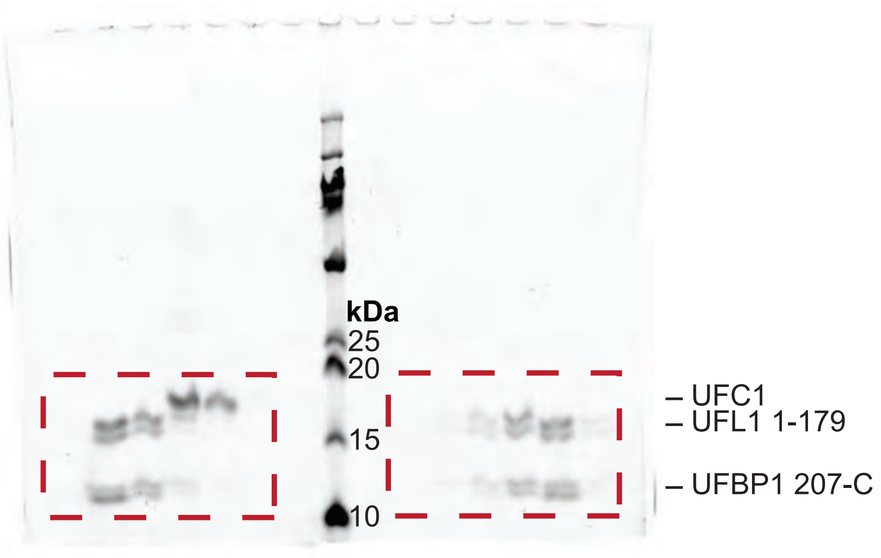
kDa
25
20
15
10
UFC1
UFL1 1-179
UFBP1 207-C

u

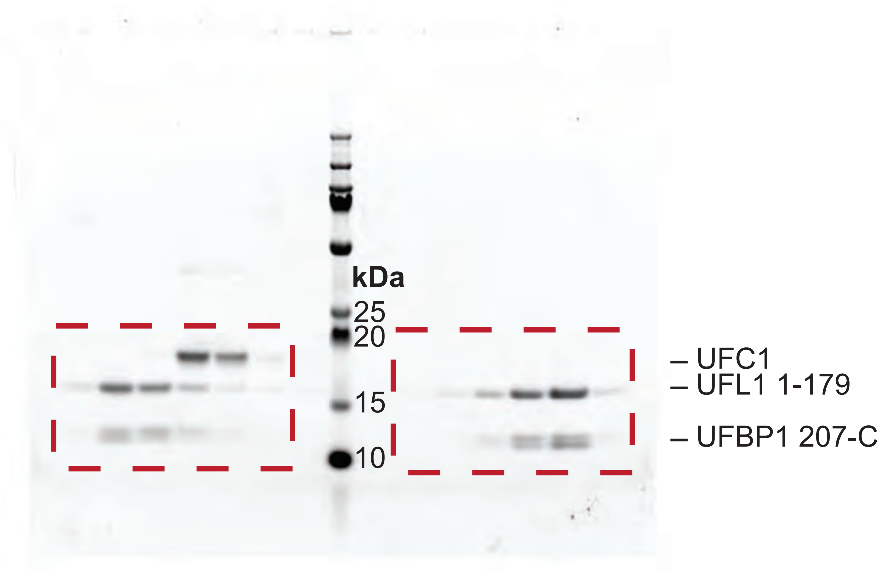
kDa
25
20
15
10
UFC1
UFL1 1-179
UFBP1 207-C

v

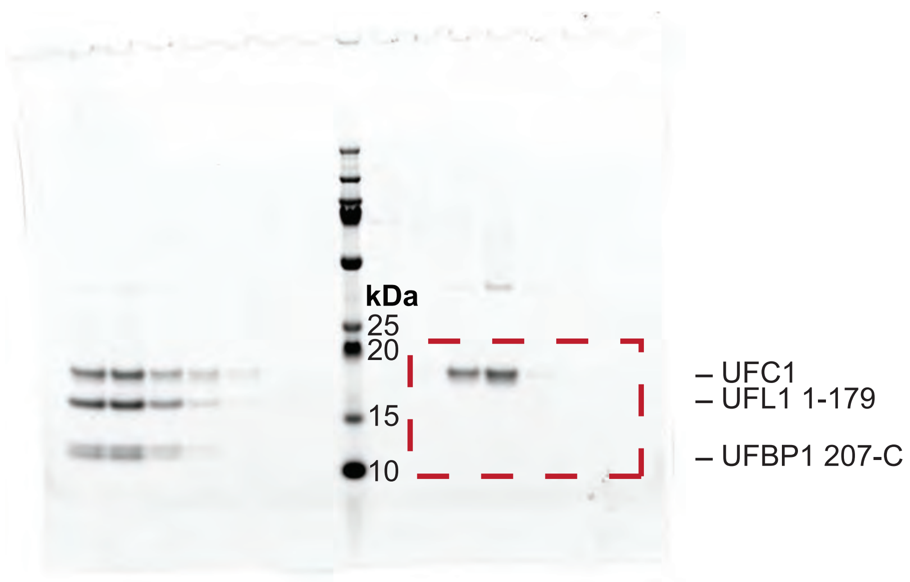
kDa
25
20
15
10
UFC1
UFL1 1-179
UFBP1 207-C

w

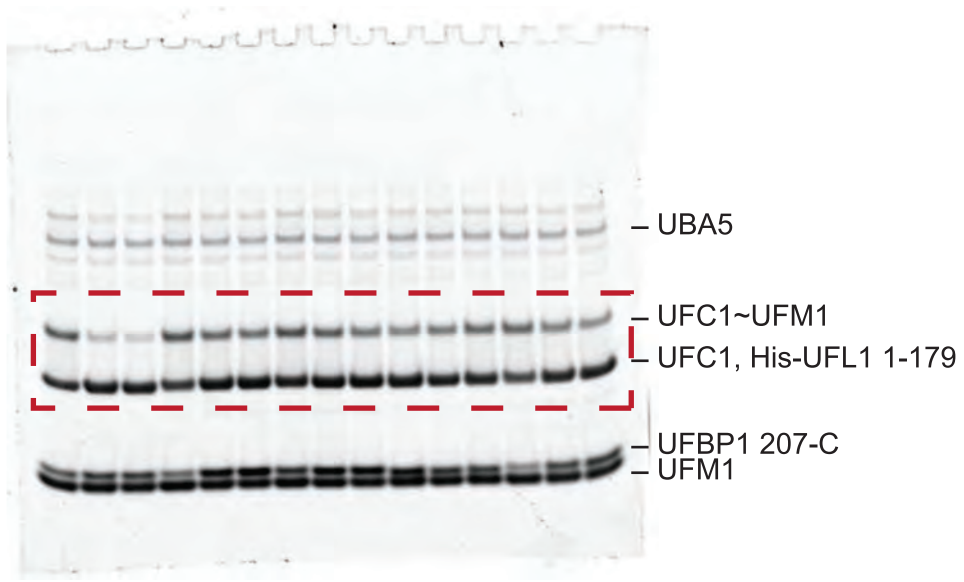
UBA5
UFC1~UFM1
UFC1, His-UFL1 1-179
UFBP1 207-C
UFM1

x

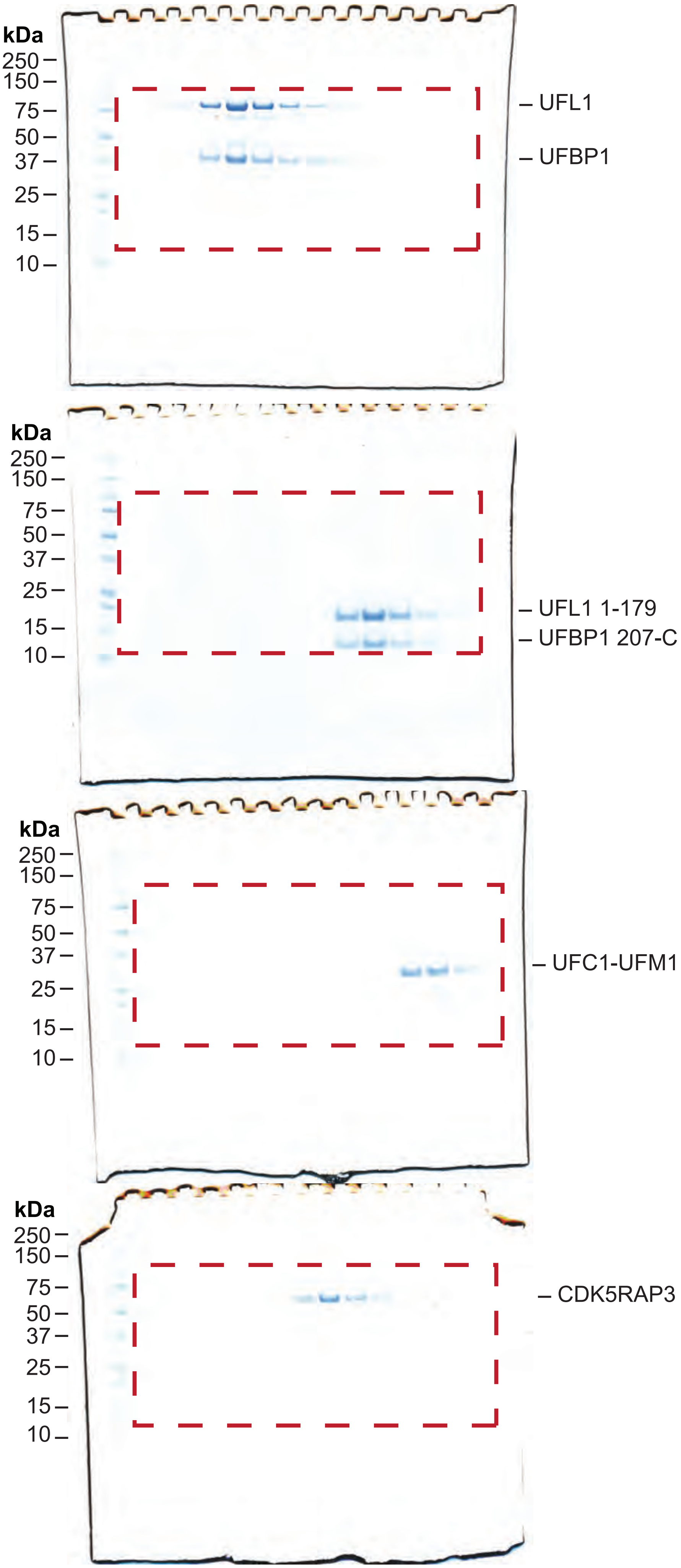
kDa
250
150
75
50
37
25
15
10
UFL1
UFBP1
kDa
250
150
75
50
37
25
15
10
UFL1 1-179
UFBP1 207-C
kDa
250
150
75
50
37
25
15
10
UFC1-UFM1
kDa
250
150
75
50
37
25
15
10
CDK5RAP3

y

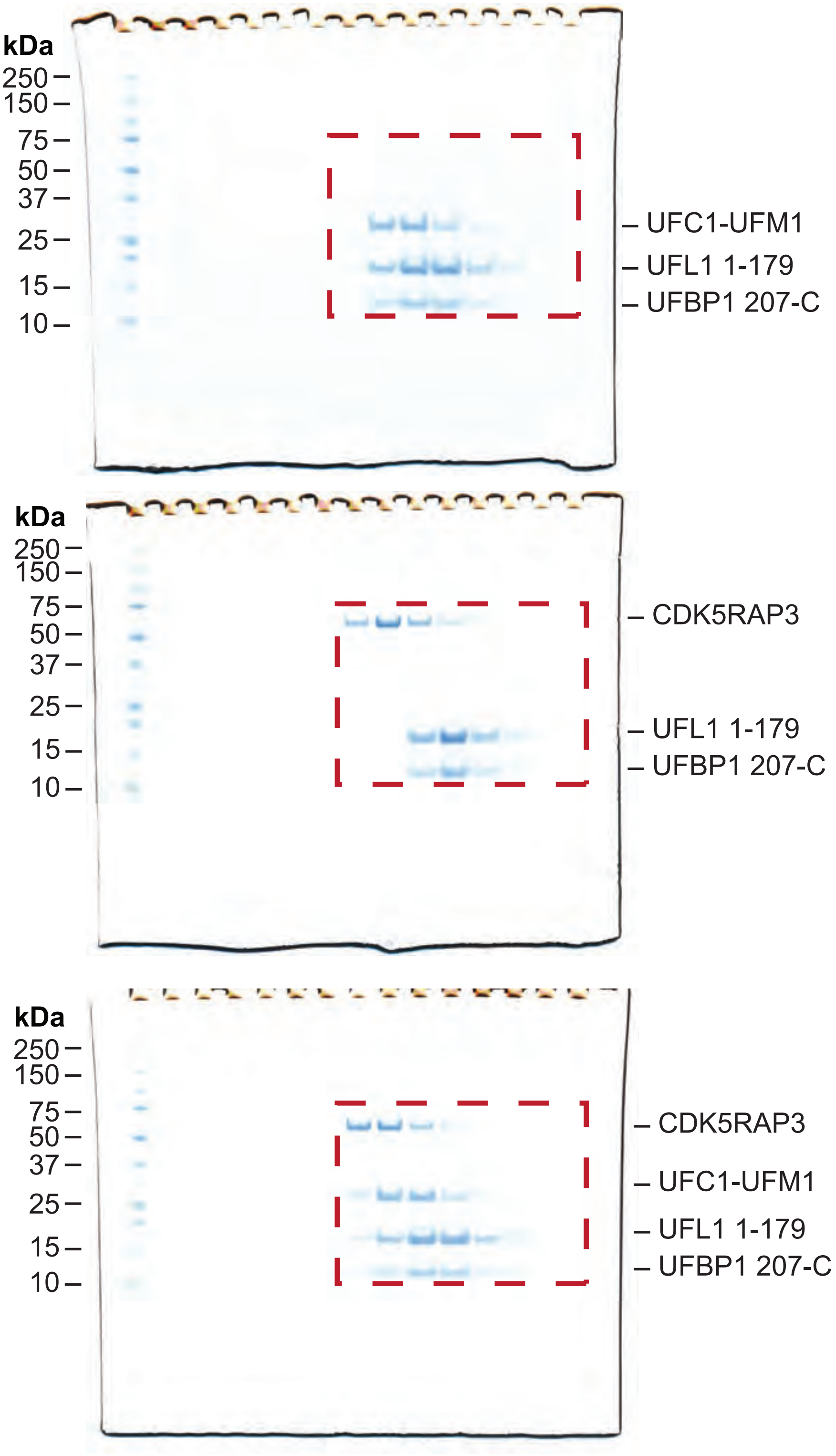
kDa
250
150
75
50
37
25
15
10
UFC1-UFM1
UFL1 1-179
UFBP1 207-C
kDa
250
150
75
50
37
25
15
10
CDK5RAP3
UFL1 1-179
UFBP1 207-C
kDa
250
150
75
50
37
25
15
10
CDK5RAP3
UFC1-UFM1
UFL1 1-179
UFBP1 207-C

z

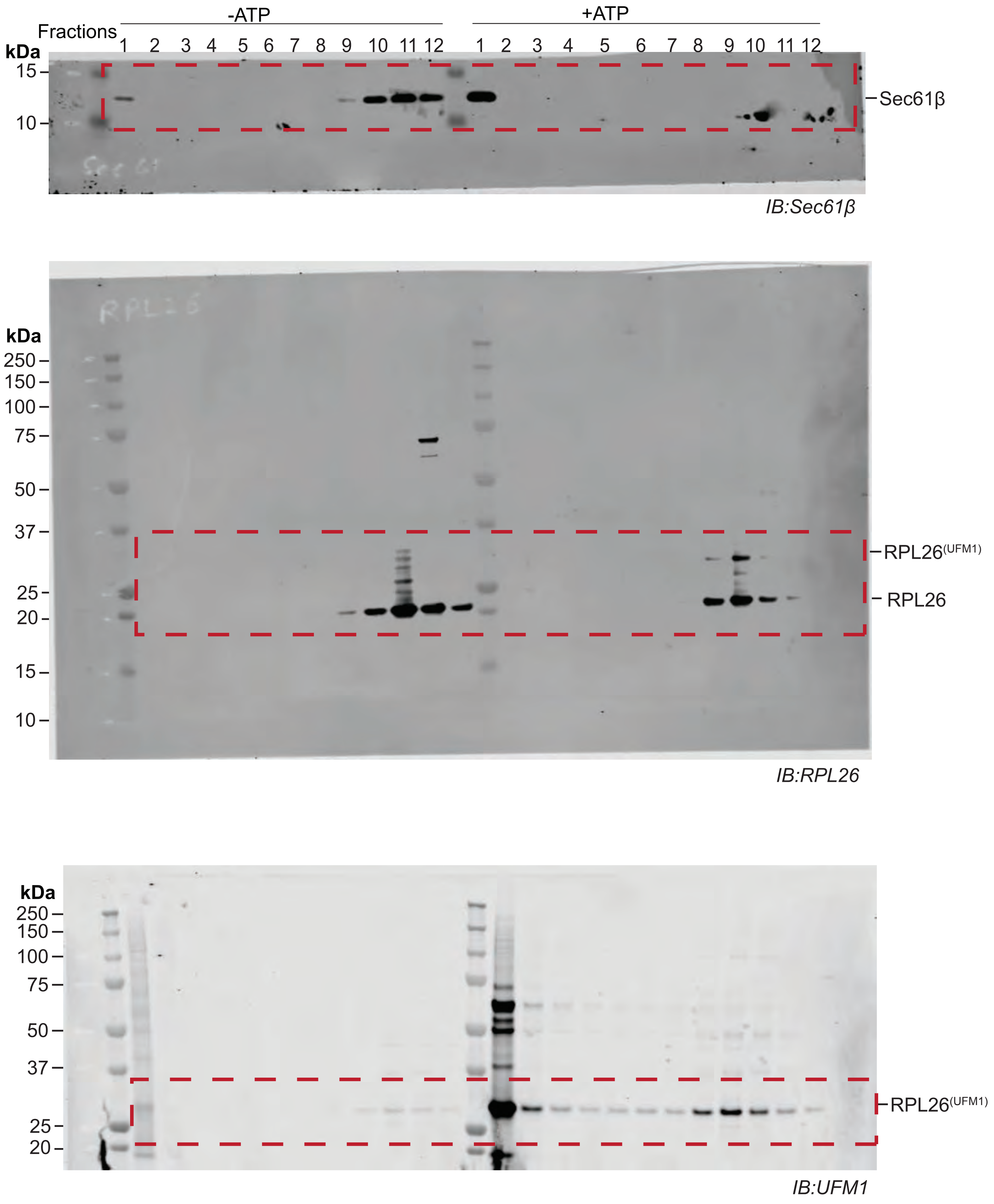

-ATP
+ATP
Fractions
1 2 3 4 5 6 7 8 9 10 11 12
1 2 3 4 5 6 7 8 9 10 11 12
kDa
15
10
Sec61β
IB:Sec61β
kDa
250
150
100
75
50
37
25
20
15
10
RPL26(UFM1)
RPL26
IB:RPL26
kDa
250
150
100
75
50
37
25
20
RPL26(UFM1)
IB:UFM1

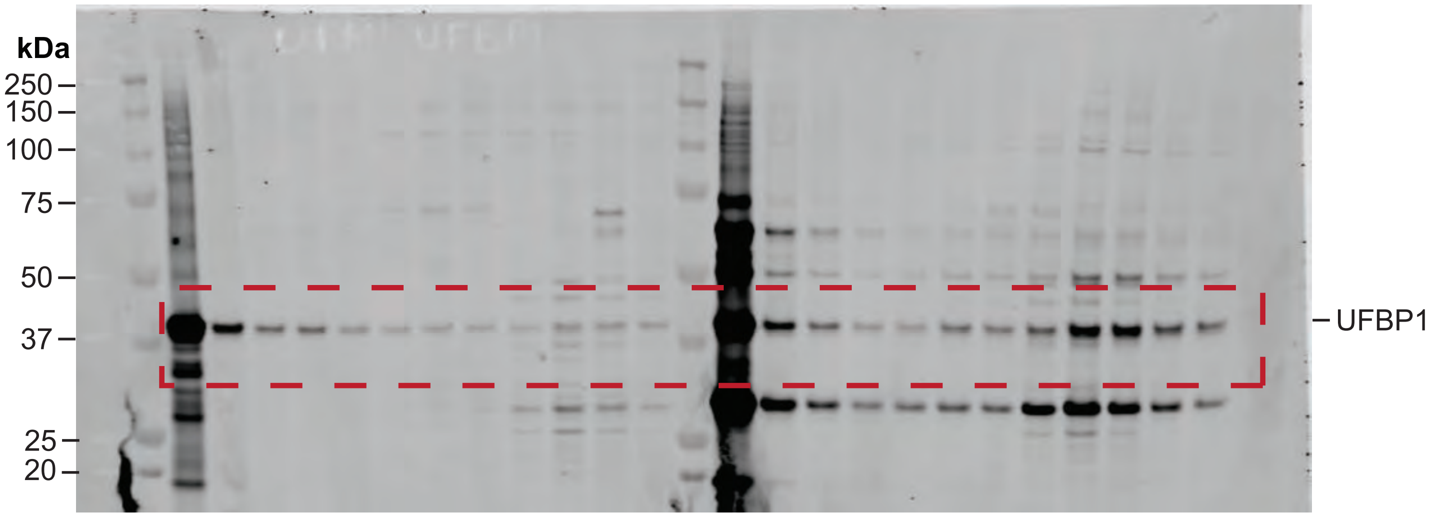

IB:UFBP1

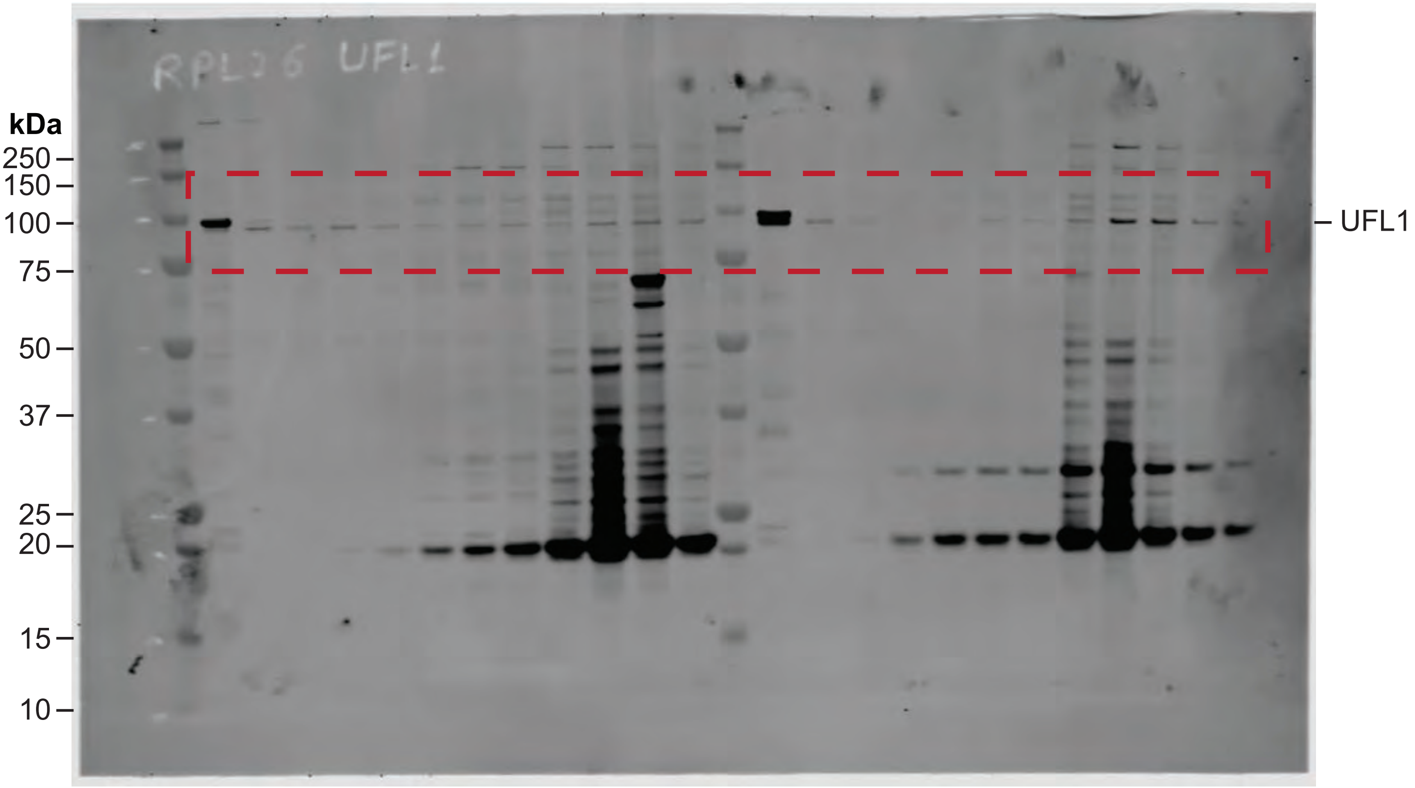

IB:UFL1

aa

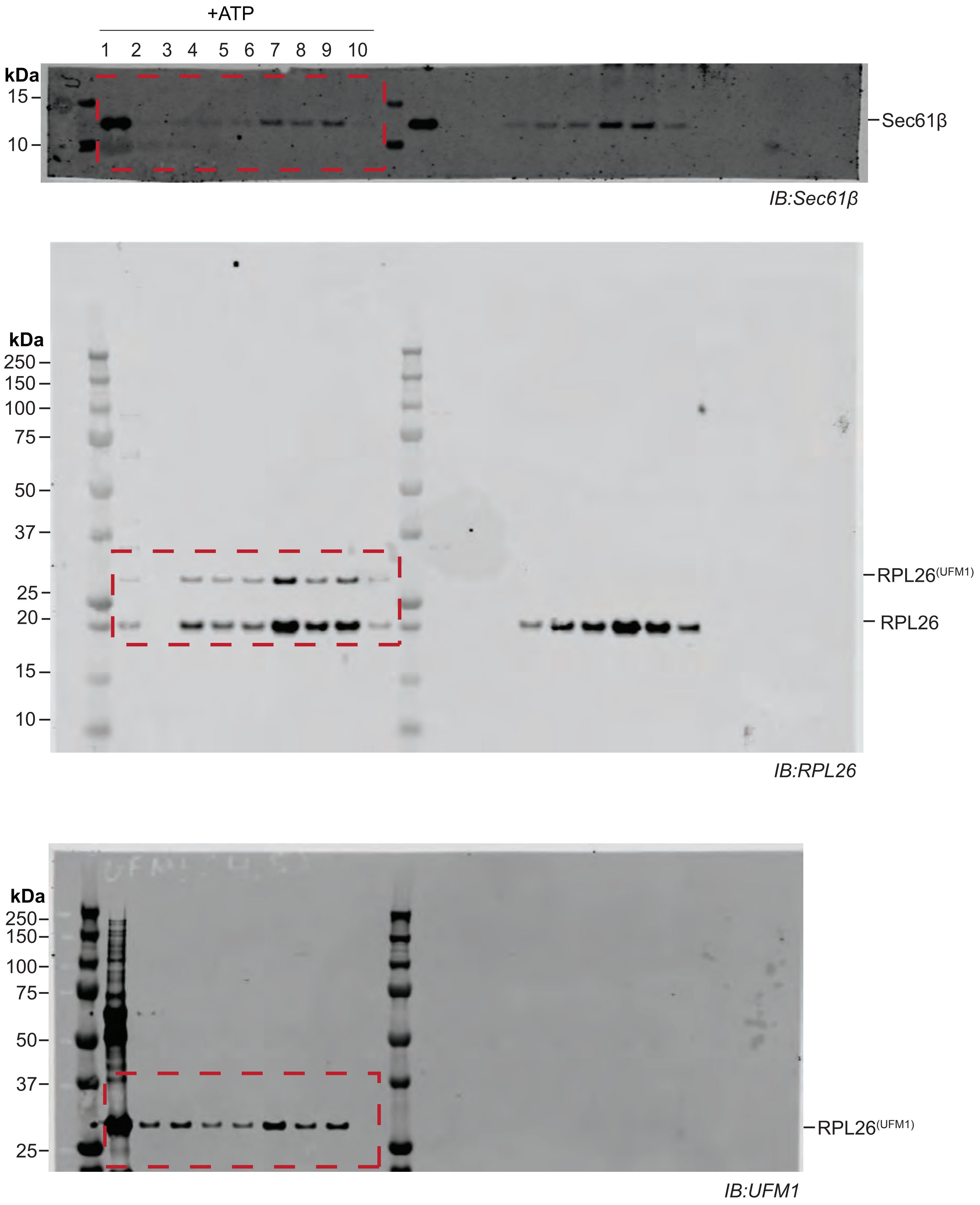
+ATP
1 2 3 4 5 6 7 8 9 10
kDa
15
10
Sec61β
IB:Sec61β
kDa
250
150
100
75
50
37
25
20
15
10
RPL26(UFM1)
RPL26
IB:RPL26
kDa
250
150
100
75
50
37
25
RPL26(UFM1)
IB:UFM1

p

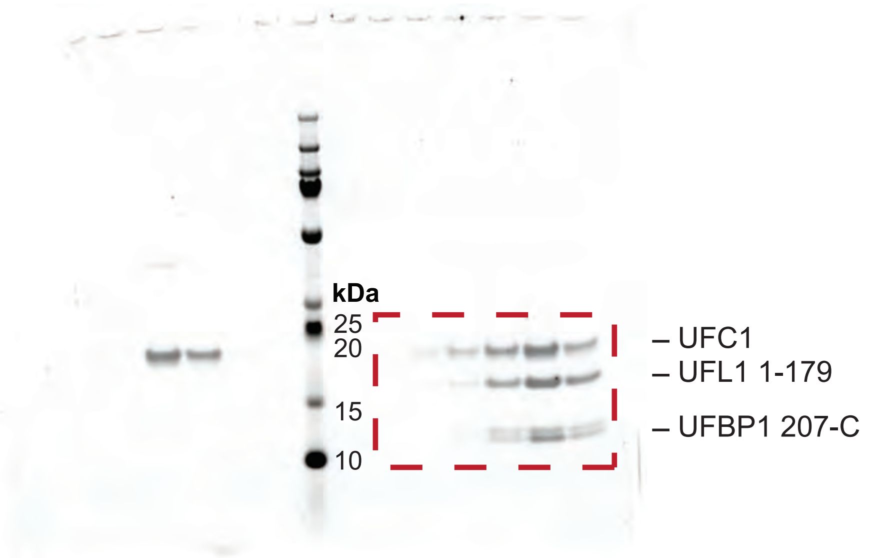
kDa
25
20
15
10
– UFC1
– UFL1 1-179
– UFBP1 207-C

q

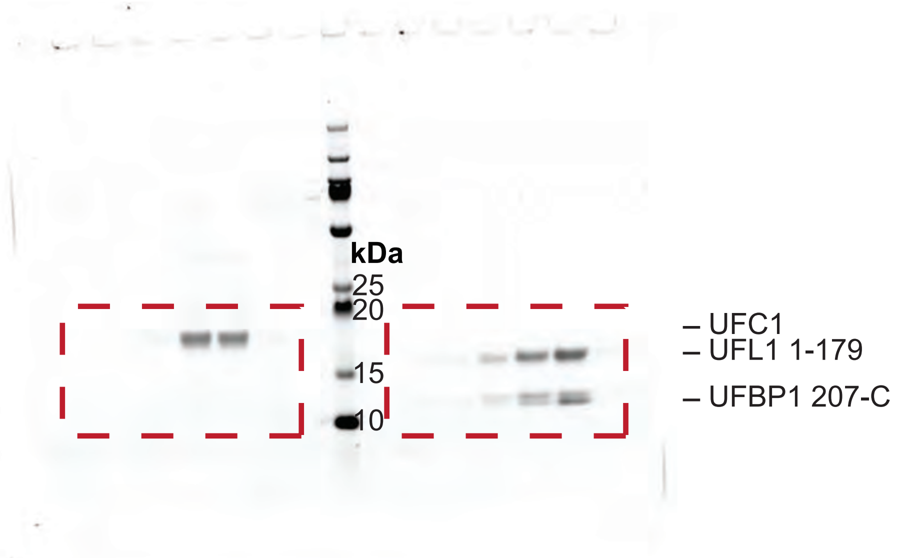
kDa
25
20
15
10
– UFC1
– UFL1 1-179
– UFBP1 207-C

r

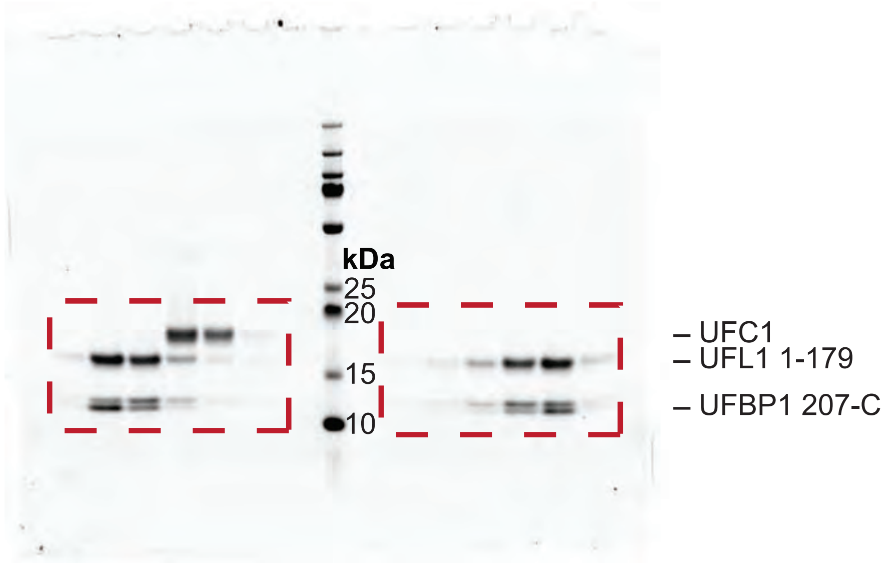
kDa
25
20
15
10
– UFC1
– UFL1 1-179
– UFBP1 207-C

ab

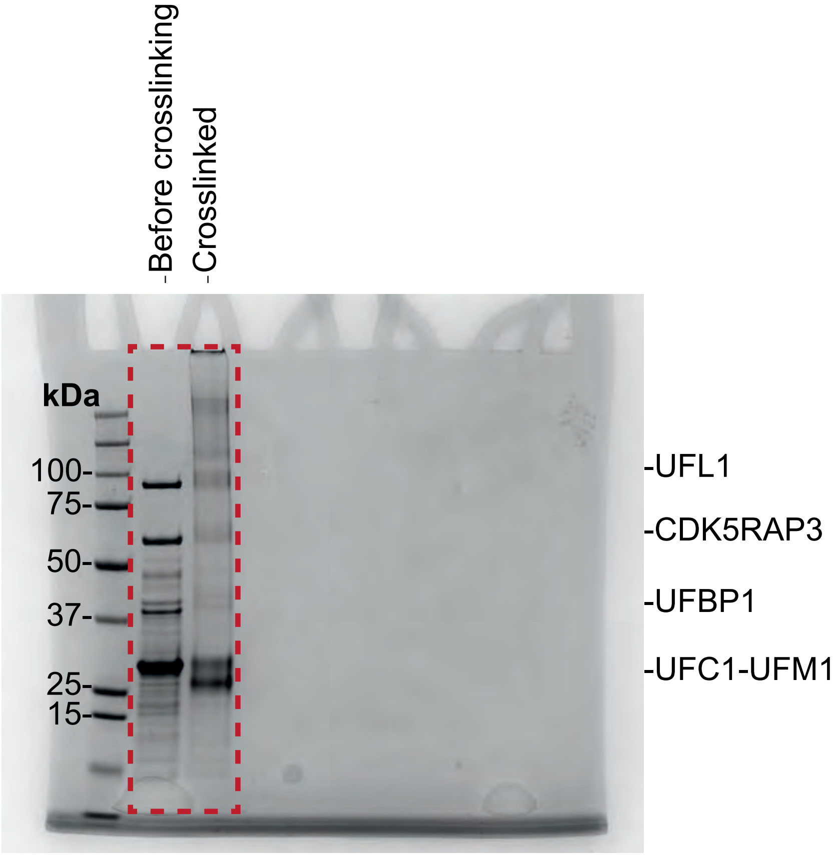

Coomassie staining
